# Age effects on Nazca booby foraging performance are largely constant across variation in the marine environment: Results from a 5‐year study in Galápagos

**DOI:** 10.1002/ece3.10138

**Published:** 2023-06-09

**Authors:** Jennifer L. McKee, Emily M. Tompkins, Felipe A. Estela, David J. Anderson

**Affiliations:** ^1^ Department of Biology Wake Forest University Winston‐Salem North Carolina USA; ^2^ Departamento de Ciencias Naturales y Matemáticas Pontificia Universidad Javeriana – Cali Valle del Cauca Colombia

**Keywords:** early‐life improvement, eastern tropical Pacific, El Niño‐southern oscillation, GPS tracking, senescence, spatial segregation

## Abstract

Foraging outcomes dictate the nutritional resources available to an organism and may vary with intrinsic factors, like age. Thus, understanding how age affects foraging performance, alone or in interaction with extrinsic factors (like environmental quality), improves our understanding of aging processes in the wild. We examined how foraging traits, measured across five breeding seasons, change with age, environmental variation, and their interaction in Nazca boobies (*Sula granti*), a pelagic seabird in Galápagos. We evaluated the hypotheses that (1) foraging performance is better in middle‐aged birds than in young ones, and that (2) foraging performance is better in middle‐aged birds than in old ones. Furthermore, favorable environmental conditions will either (3) attenuate age differences in foraging performance (by relieving constraints on young, inexperienced and old, senescent age classes), or (4) accentuate age differences (if middle‐aged birds can exploit abundant resources better than other age classes can). Incubating birds tagged with GPS loggers (*N* = 815) provided data on foraging performance (e.g., total distance traveled, mass gained) to evaluate interactions between age and environmental variation (e.g., sea surface temperature). Poor environmental conditions associated with the cool phase of the El Niño‐Southern Oscillation increased foraging effort, including foraging distance and duration, for example. Across age classes, foraging boobies responded similarly to environmental variation except for female mass gain rate: age‐related declines in mass gain rate were reduced under favorable environmental conditions. Birds of different ages also searched in somewhat distinct areas in the poor conditions of 2016, but not in other years. In several foraging traits, including foraging duration and distance, female boobies showed predicted early‐life improvement and late‐life decline, following the established pattern for reproductive traits in this species. Thus, deficits in resource acquisition (this study) may contribute to the poor survival and reproductive outcomes previously observed in old Nazca boobies, particularly in females.

## Graphical abstract

Foraging performance affects the nutritional resources available for reproduction and survival, influenced by the environment and an animal's individual factors, like age. We evaluated how foraging traits, measured across 5 years, changed with age, environmental variation, and their interaction in Nazca boobies, a tropical, pelagic seabird in Galápagos. Although the 5 years encompassed significant environmental variation, including an El Niño event, foraging boobies responded similarly to environmental variation across age classes, except for foraging location and female mass gain rate.

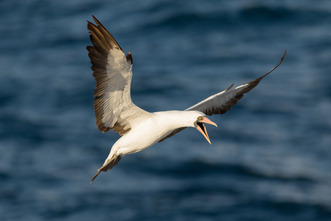

## Resumen

Los resultados del forrajeo determinan los recursos nutricionales disponibles de un organismo, los cuales pueden variar por factores intrínsecos, como la edad. Por lo tanto, el entendimiento de cómo la edad afecta el rendimiento de forrajeo, sola o en interacción con factores extrínsecos (como la calidad ambiental), mejora nuestro entendimiento de los procesos del envejecimiento en la naturaleza. Nosotros examinamos como las características de forrajeo, a lo largo de cinco temporadas reproductivas, cambian con la edad, la variabilidad ambiental y sus interacciones en el Piquero de Nazca (*Sula granti*), un ave marina pelágica en las Islas Galápagos. Evaluamos las hipótesis que (1) el desempeño de búsqueda de alimentación es mejor en aves adultas que en las jóvenes, y que (2) este desempeño es mejor en aves adultas que en las viejas. Además, las condiciones ambientales favorables (3) atenuaran las diferencias de edades en el rendimiento de forrajeo (aliviando las restricciones en los jóvenes, inexpertos, y viejos), o (4) acentuaran las diferencias de edad (sí los adultos pueden explotar recursos abundantes de mejor forma que otros grupos etarios). Colocamos dispositivos GPS en aves incubadoras (*N* = 815), para colectar datos sobre el rendimiento del forrajeo (p. ej. distancia total de viaje, incremento de peso) para evaluar las interacciones entre edad y la variación ambiental (p. ej. temperatura superficial del mar). Las condiciones ambientales adversas relacionadas a la fase fría de El Niño Oscilación del Sur incrementaron el esfuerzo de forrajeo (la distancia de forrajeo y su duración, por ejemplo). En todos los grupos etarios, los piqueros respondieron de forma similar a la variación ambiental, excepto la tasa de ganancia de peso de las hembras. Las reducciones relacionadas a la edad en la tasa de ganancia de peso fueron menores durante condiciones ambientales favorables. Las aves de diferentes grupos etarios buscaron alimento en zonas levemente diferentes durante las condiciones adversas de 2016, pero no en otros años. En varias características de forrajeo, incluyendo la duración y la distancia, las hembras mostraron un esperado mejoramiento en los primeros años y un declive en su vida tardía, mostrando el patrón establecido en rasgos reproductivos en esta especie. Por lo tanto, el déficit en la adquisición de recursos (este estudio), puede contribuir a la reducida supervivencia y los resultados reproductivos previamente observados en piqueros de Nazca viejos, especialmente en las hembras.

## 1 INTRODUCTION

Acquiring food (“foraging”) is a central activity in any animal's life, providing the nutritional resources available to the organism for activities affecting reproduction and survival. Foraging performance can be expected to vary with the forager's circumstances, including its age. A variety of traits are now known to vary with age in wild organisms, including age‐related improvement in physiology, muscle strength, and breeding outcomes in young adulthood, superior performance in middle age, and senescent decline in old age (Lemaître & Gaillard, 2017; Nussey et al., 2013). However, despite its central role in an animal's life, age‐related changes in the ability of an organism to meet its energy needs are poorly understood, particularly in late life. In studies done on seabirds, individual foraging ability shows age‐related variation in some cases (e.g., Breed et al., 2011; Frankish et al., 2020; Galbraith et al., 1999; Lescroël et al., 2023; Rutz et al., 2006), but not all (Elliott et al., 2015; Froy et al., 2015), providing an unclear picture of the roles of accumulating experience and other benefits of age versus that of advancing physiological decline for foragers.

Long‐lived, iteroparous species, including many seabirds, will encounter substantial environmental variation during their lifespan, and life‐history theory predicts a flexible response (Erikstad et al., 1998; Stearns, 1992) conditioned on factors (like age) to optimize performance (Kirkwood & Rose, 1991; Stearns, 1992; Williams, 1957). Thus, age might interact with environmental quality to affect performance (how capably an organism meets its needs), including for foraging (e.g., Daunt et al., 2007, Limmer & Becker, 2009). Empirically, two patterns are observed. In one, poor environments enhance age effects because the performance of young and/or old animals suffers more than that of middle‐aged animals, due to inexperience or senescence, respectively (e.g., for age at first breeding, clutch size, hatching success, and fledgling success; Boekelheide & Ainley, 1989, Sydeman et al., 2001). In the other, good environmental conditions (not poor ones) enhance age effects because middle‐aged animals are better able to take advantage of abundant resources than other age classes are (e.g., in young versus experienced seabirds for foraging efficiency; Daunt et al., 2007, and meal mass; Limmer & Becker, 2009).

Few studies have evaluated these age‐by‐environment interactions on foraging in any wild animal (but see Daunt et al., 2007, Limmer & Becker, 2009), but doing so improves our understanding of the aging process (Reed et al., 2008; Tompkins et al., 2017; Weimerskirch, 1992) and population responses to environmental change (Coulson et al., 2001). Here we evaluate interactions between age and environmental variation on foraging performance in known‐age, incubation‐stage Nazca boobies (*Sula granti*), a tropical, pelagic seabird that plunge‐dives for fish prey on trips that may span 100s of kilometers (km) over several days (Howard et al., 2021; Zavalaga et al., 2012). We first evaluate age effects, and then age‐by‐environment interactions on measures of foraging performance (trip distance and duration, searching time, mass gained, and rate of mass gain), separately by sex. An earlier two‐year study found no old‐age decline in foraging trip length and foraging efficiency (rate of mass gain; foraging distance and searching time were not examined: Howard et al., 2021). Here, we enhance the power of inference with a larger set of foraging traits measured over 5 years that encompass oceanographic variation across the El Niño‐Southern Oscillation spectrum in the eastern Pacific Ocean.

In seabirds, foraging efficiency (Fayet et al., 2015) and body condition (Weimerskirch, 1992) often improve with age and experience in naïve seabirds during an extended sub‐adult phase. But recruitment to the breeding population poses further challenges. In early life, foraging performance (such as foraging trip duration and the amount of food delivered to nestlings) generally improves with age and experience (e.g., Daunt et al., 2007; Frankish et al., 2020; Galbraith et al., 1999; Lescroël et al., 2019, 2023; Limmer & Becker, 2009). Early‐life improvements in foraging ability may result from increasing experience (e.g., Daunt et al., 2007) or from increasing physiological competence with age (e.g., stronger bite force in middle‐aged versus young female Nazca boobies; Rebol & Anderson, 2022). Compositional changes in an age cohort may also contribute to observed early‐life improvement at the population level if poor‐quality individuals die young (e.g., Barbraud & Weimerskirch, 2005).

In late life, evidence of foraging senescence varies across studies. In some populations, old seabirds took longer foraging trips (Catry et al., 2006; Frankish et al., 2020; Lecomte et al., 2010), gained less mass per day (Catry et al., 2006), displayed lower activity (Catry et al., 2011), and fed nestlings less often than middle‐aged birds did (Galbraith et al., 1999), while senescence was not detected in other studies (Elliott et al., 2015; Froy et al., 2015; Lescroël et al., 2019). Space use can also vary by age (Lecomte et al., 2010; Navarro et al., 2010) or by sex (Clark et al., 2021; Clay et al., 2018) because of competitive exclusion, different dietary requirements, or shifting optimal foraging strategies. Significant challenges can obscure senescent decline. For example, foraging studies may depend on cross‐sectional data covering a small number of breeding seasons, acknowledging the difficulty of measuring characteristics expressed 10s–100s of km from the breeding colony (typically using small data loggers) over the entire lifespan in a longitudinal study. Under cross‐sectional approaches, early mortality of low‐quality phenotypes can increase the average quality (and thus average performance) of a cohort with age, hiding senescence (Cam et al., 2002). Also, where age‐by‐environment interactions exist, age effects may be minor if the range of environmental variation is limited to conditions associated with minimal differences among age classes.

Our study of age‐by‐environmental effects on foraging builds on earlier studies of Nazca boobies. Adults return to the study colony as potential breeders at 3–7 years of age (Maness & Anderson, 2013). Reproductive performance improves through ages 6–10, is high through middle age, and then survival and reproduction decline from the mid‐ to late‐teens onward (actuarial and reproductive senescence, respectively; Anderson & Apanius, 2003, Tompkins & Anderson, 2019, 2021). Corresponding with these age‐related changes in reproduction and survival, bite force is lower in young and old Nazca booby females than in middle‐aged ones, and declines in very old age in males (Rebol & Anderson, 2022), implicating physiological decline as a cause of reproductive and actuarial senescence. Marked sex effects on aging are apparent: due to a male‐biased adult sex ratio and obligate bi‐parental care, females breed more frequently than males do (Maness & Anderson, 2007; Townsend & Anderson, 2007), show enhanced reproductive and actuarial senescence compared to males (Tompkins & Anderson, 2019), and lose bite force earlier in old age than males do (Rebol & Anderson, 2022). Environmental effects on aging are also evident in some measured traits. For example, in a challenging environment breeding probability and breeding date suffer more in young breeders than in middle‐aged breeders (Tompkins & Anderson, 2021). However, we know little about age‐by‐environment interactions on foraging traits in Nazca boobies, a notable gap in our understanding of how variation in demographic performance develops across the lifespan and in response to environmental quality.

Across the five breeding seasons of this study, we use foraging breeders during their egg‐incubation period to examine age‐by‐environment interactions for the rate of mass gain and the underlying foraging traits (mass gain, foraging duration; following Howard et al., 2021), and, for the first time, for foraging location, distance traveled, and time spent searching. We evaluate age‐by‐environment interactions with respect to a population‐level shift in breeding date (Tompkins et al., 2017) and two environmental predictors linked to the El Niño‐Southern Oscillation (ENSO; sea surface temperature and cloud cover; Table 1). Using patterns of observed age effects in breeding traits as models (Tompkins et al., 2017; Tompkins & Anderson, 2019, 2021), and assuming a tight link between foraging and breeding performances, we predict that: (1) foraging performance will be better in middle‐aged birds than in young ones in our cross‐sectional sample (higher rate of mass gain, larger mass gain, and shorter foraging duration, distance traveled, and time searching in middle‐aged birds) and (2) foraging performance will be higher in middle‐aged birds than in old ones, with lower performance beginning in the mid‐teens. In a second analysis stage, we evaluate age‐by‐environment interactions for foraging traits. Favorable environmental conditions associated with high resource availability are predicted to improve foraging performance (increase mass gain and the rate at which mass is gained, and decrease foraging duration, distance traveled, and time spent searching) and may either (3) attenuate age differences in foraging traits or (4) accentuate age differences in foraging traits (if middle‐aged birds can best exploit high‐quality environments; Hernández et al., 2015, Oro et al., 2014). Spatial separation of age classes may also result from the same factors predicted to drive age‐related changes in foraging efficiency. For example, changes in physiology or motivation may lead to age‐specific optimal foraging locations or competitive exclusion of young and/or old birds from some areas (Lecomte et al., 2010). Differences between sexes are examined, acknowledging past results linking sex to age‐related changes in performance (Rebol & Anderson, 2022; Tompkins & Anderson, 2019, 2021), and clear differences between the sexes in body size (females are 17% heavier) and flight characteristics (Howard et al., 2021).

**TABLE 1:** Descriptions of fixed‐effect predictors (other than age) used to model Nazca booby foraging traits.

Analysis	Predictor(s)	Variable definition	Justification for inclusion and/or role in analyses
Stage 1	Breeding Season	Each breeding season spans two calendar years and was labeled by the first calendar year.	Breeding Season was used in the Stage 1 analyses to control large‐scale environmental variation.
Stage 1/2	Date	Expressed as daily increments across November–January of each breeding season; standardized.	Date influenced foraging outcomes in Howard et al. (2021), and so appears in all models to control seasonal variation in foraging.
Stage 1/2	Logger	Two GPS logger models were deployed on Nazca boobies (i‐gotU® GT‐120, GT‐600).	Differences in logger size can influence foraging in seabirds (Clark et al., 2022). Logger appeared in all models to control the combined effects (on foraging performance) of differences between the GT‐120 and GT‐600 units in size, mass, and attachment location.
Stage 1/2	Wing Loading	Body weight at departure (9.8⁎kg) divided by wing area (m^2^); standardized.	Wing loading incorporates a bird's structural body size and mass at departure, affecting the production of thrust and lift (Heerenbrink et al., 2015). Wing Loading = (9.8⁎body mass)/(0.04 + 0.95⁎wing chord)^2^.
Stage 2	Sea Surface Temperature (SST)	SST (°C) averaged over the foraging area and time‐matched to each foraging absence. Residual SST was used (see Supplementary Material).	During egg laying, Nazca booby performance (breeding date, clutch size) is relatively high during an El Niño and relatively low during La Niña (Tompkins & Anderson, 2021). Residual SST indicates whether the sea surface temperature is relatively warm or cool for a given time of year.
Stage 2	Median Breeding Date (of the population)	The median clutch initiation date in a comprehensively monitored colony subsection; standardized.	Population level shifts in breeding date may reflect changing food availability. The 5 years of this study span a 1‐month shift in the timing of breeding (Tompkins & Anderson, 2021).
Stage 2	Cloud Cover	Proportion of each 0.75‐degree latitude‐longitude grid square covered by clouds within 2 km of Earth's surface.	Ambient light level, affected by cloud cover, can influence the type of prey captured and foraging success in seabirds (Elliott & Gaston, 2015, Regular et al., 2011).

Although the current study is largely cross‐sectional (see Methods), longitudinal studies in Nazca boobies have found little scope for selective disappearance to bias age effects for several elements of reproductive performance (Tompkins & Anderson, 2019, 2021), supporting this approach. A study during the incubation period reduces the possibility of selective sampling inherent to studies during the chick‐rearing period: low‐quality birds that failed during incubation may be under‐represented during chick‐rearing, reducing the range of response variables during chick rearing and obscuring the true effects of age and environment on performance.

## 2 METHODS

### 2.1 Study site

We studied foraging traits of Nazca boobies breeding at Punta Cevallos on Isla Española, Galápagos (1°23′ S, 89°37′ W; Apanius et al., 2008, Huyvaert & Anderson, 2004 give details of the site). Nazca boobies breed seasonally at Punta Cevallos, laying eggs in October–January (Tompkins & Anderson, 2021), and most nestlings reach independence by the following June. Each breeding season spans two calendar years; we refer to the entire breeding season by the first calendar year (i.e., the 2011–2012 breeding season as “2011”) and enumerate date with an extended Julian calendar (i.e., 1 January 2012 in the “2011” breeding season is day 366). Egg incubation starts immediately after laying and pair members alternate incubation with time spent foraging. Unique leg bands identify individuals at Punta Cevallos and annual banding of nestlings started in 1984, providing known‐age individuals. Sex‐specific adult voices reliably indicate sex (Maness & Anderson, 2007).

### 2.2 Tagging methods

Foraging data were collected from incubating boobies early in the breeding cycle, from November–January, over five breeding seasons (2011–2012, 2014–2016). GPS loggers were deployed on 815 unique breeding individuals (1179 separate deployments), with 266 birds tagged in two or more seasons. We tagged banded birds in four age groups (“AgeGroups”) that match the observed age‐dependent pattern of reproductive success (Anderson & Apanius, 2003; Tompkins & Anderson, 2019): “Young” (low success, 4–9 years), “Middle Age” (peak success, 11–16), “Old” (low success, 17–20), and “Oldest” (negligible success, 21–25). No birds between 26 and 28 years, the maximum known lifespan, bred to include in this study. Age is known precisely for the majority of tagged birds (98% were banded as nestlings). For the minority banded as adults (15 birds, all in the Old group), age was estimated based on the assumption that birds were 4 years old when banded (the median recruitment age, Maness & Anderson, 2013). To reduce temporal environmental noise, loggers were deployed synchronously on same‐sex groups of 3–4 incubating adults, including one member of each age group (whenever possible based on the availability of known‐age incubators).

Two different i‐gotU® GPS loggers (Mobile Action Technology, Taiwan) were used during the study period: the GT‐120 loggers were deployed in all five breeding seasons and the GT‐600 loggers in 2014, 2015, and 2016. The i‐gotU® GT‐120 and GT‐600 GPS loggers were attached following Howard et al. (2021), and approximately evenly across the age classes and sexes (Table S1). We taped the smaller GT‐120 loggers under the tail to minimize drag during plunge dives, and the larger GT‐600 loggers above the tail to avoid contact with rocks when the birds walked on land. Loggers were deployed on the second or third day of a bird's incubation bout (incubation bouts lasted 2–7 days during the study), at nests established ≥7 days earlier. Daily nest monitoring provided clutch initiation dates and incubation schedules. GPS loggers were attached and removed at the nest, with removal after 5–10 days and at the completion of at least one absence >3 h. We weighed birds at logger deployment and retrieval and measured flattened, stretched wing chord (wrist to wing tip) during retrieval. In the case that a GPS logger collected more than one foraging trip from a bird, we used only the first trip in analyses. Some individuals (33%) were sampled in more than one breeding season. The Supplementary Material contains further details of logger type, deployment, and sample sizes (Table S1).

### 2.3 Foraging traits

We evaluated age effects on five foraging response variables (hereafter, “foraging traits”): the duration of a foraging absence, or the time a bird was absent from the nest to forage (“Absence Duration”; hours), mass gained during the foraging absence (“Mass Gain”; grams [g]), the ratio of mass gained to time absent (“Mass Gain/hour”; g/hr), the summed distance between consecutive locations (“Total Distance”; km), and the duration of time spent actively searching for and acquiring prey (“Time Searching”; hours). Additionally, we compared core foraging areas among age groups to identify age differences in foraging location (described in “Spatial Segregation” below). We measured our response variables over a foraging absence (the period between ending one incubation shift and starting the next), and not over a foraging trip (the period between departing and returning to the colony) because some birds take multiple short foraging trips (returning to the colony, but not to the nest) during one absence (note that absence duration and trip duration are highly correlated; *r* = .85, df = 909, *p* < .01). Variables measured over a foraging absence thus better capture the total effort (e.g., Absence Duration, Total Distance) and total payoff (e.g., Mass Gain) that the bird achieves within the constraints imposed by the incubation schedule. We did not evaluate the maximum distance from the colony because it was highly correlated with Total Distance (*r* = .91, df = 1009, *p* < .001). Absence Duration, Total Distance, and Time Searching were calculated using daylight periods only because Nazca boobies rarely forage (Zavalaga et al., 2012; Figure S1) or fly at night (Figure S3). Mass Gain was calculated by subtracting mass at departure (calculated from mass at logger deployment and predicted rates of daily mass loss during incubation; see Supplementary Material) from mass at the completion of the Absence Duration. Mass Gain/hour was the mass gained (Mass Gain) divided by the total foraging duration, including daylight and nighttime periods. Total Distance and Time Searching were calculated from each bird's sequence of logger locations during a foraging absence. Distance and speed are sensitive to the sampling frequency of GPS loggers when using traditional straight‐line displacement measurements (Noonan et al., 2019), so we interpolated data from the 2014–2016 breeding seasons (collected at 3‐min intervals) to 5‐min intervals to match the sampling frequency of the 2011 and 2012 breeding seasons.

Before calculating Time Searching, activities during the foraging absence were classified for each sex using the Expectation–Maximization binary Clustering (EMbC) algorithm in the R package *EMbC* (Garriga et al., 2016). Because of known sex differences in this species in flight characteristics (Howard et al., 2021) and age‐related performance (Rebol & Anderson, 2022; Tompkins & Anderson, 2019), we classified activities separately for males and females. EMbC incorporates the speed and turning angle from successive logger locations to discriminate four activity states: resting (low speed/low turning angle), level flapping flight or commuting (high speed/low turning angle), relocating (high speed/high turning angle), and localized searching (low speed/high turning angle). In a two‐step process (see Supplementary Material), EMbC estimated the threshold for separating low and high speed to be below 5.8 m/s for males and 7.12 m/s for females, and the threshold for separating low and high turning angles to be less than 0.74 radians for males and 0.34 radians for females (Table S3). Time Searching was calculated as the sum of the time spent doing localized searching (from EMbC) during each Absence Duration. We performed all data processing and statistical analyses in R v. 4.0.2 (R Core Team, 2021).

### 2.4 Statistical analyses

We used linear mixed models (LMMs; *lme4* R package; Bates et al., 2015) in an information‐theoretic approach to evaluate the influences of age and environment on foraging traits. Analyses were conducted in two stages. In Stage 1, we found the best age function describing variation in the foraging traits. The top age parameterization from Stage 1 was passed to Stage 2, where we described variation in the foraging traits by age, environmental variation, and their interaction. Response variables were analyzed separately by sex, motivated by sex‐specific effects of age on Nazca booby breeding performance (Tompkins & Anderson, 2019) and bite force (Rebol & Anderson, 2022).

#### 2.4.1 Stage 1: Patterns of aging

In Stage 1, we evaluated age as a predictor of foraging performance in Nazca boobies and found the best‐supported function relating age to each response variable (Mass Gain/hour, Mass Gain, Absence Duration, Total Distance, and Time Searching). Age (“Age”, used as a continuous predictor) of sampled birds spanned 4–25 years (for males; 4–24 years for females), with all ages except 10 appearing in the data. Performance of models omitting Age was compared to that of models with linear, linear + quadratic, or threshold parameterizations of Age. “Two‐threshold” models divided the lifespan into three periods—early, middle, and late life—and fit linear changes with Age within each period (see Supplementary Material for model parameterization details). The boundaries of each period were determined by the threshold position(s), which varied across candidate models. We evaluated early‐life thresholds at ages 6–8 for females and at ages 7–10 for males, and late‐life thresholds at ages 14–17 for both sexes (based on observed aging patterns in reproductive traits; Tompkins & Anderson, 2019, 2021). We also evaluated models containing a single threshold (testing for early‐life improvement in the absence of late‐life decline or vice versa). Additionally, single‐threshold models considered thresholds falling at ages 12 and 13. We fit 29 models for males and 24 models for females.

All Stage 1 models included additional predictors of “Breeding Season” (a five‐level factor), extended Julian date (“Date”, controlling seasonal changes), logger type (“Logger”, controlling any effect of the different weights and/or attachment locations of the GT‐120 vs. GT‐600 models), and a measure of structural size (“Wing Loading”; Table 1). Thus, a model including Breeding Season, Date, Logger, and Wing Loading served as our “null” (simplest) model and was compared to candidate models including age effects (plus these four predictors) to evaluate the importance of age. All models also included crossed random effects of individual identity (“ID”) and of a 10‐day binned window of foraging departure dates (“DepartGroup”) to control temporal environmental variation. Some individuals were sampled repeatedly in different breeding seasons, explaining the inclusion of ID (28% of individuals in the Absence Duration dataset were sampled more than once, for example). Models were ranked using AIC‐based model selection (AICc to correct for a small sample size; Burnham & Anderson, 2010). The model explaining variation in the data best had the lowest AICc value (“top model”). We discuss model selection uncertainty but did not model‐average our coefficient estimates (by AICc model weights) in cases where multiple candidate models receive strong support (falling within ∆2 AICc of the top model), under the following reasoning. Our model set includes predictors whose effects are understood by simultaneously interpreting two or more regression coefficients (a quadratic age function, threshold age parameterizations, and in Stage 2, interaction terms), where the meaning of individual regression coefficients changes across models depending on the presence/absence of higher order terms (e.g., for polynomials or interactions) or according to threshold age position (for threshold parameterizations). In this circumstance, averaging coefficient estimates across models is not appropriate (Cade, 2015). We used the Age parameterization from the top model in Stage 1 in Stage 2 analyses. If the null model from Stage 1 (no Age predictor) had the lowest AICc, then Age was not included in Stage 2.

#### 2.4.2 Stage 2: Age by environment

In Stage 2, alongside Age (if appearing in the top model within Stage 1), we evaluated the effects of three environmental predictors on foraging response variables (Table 1). Inter‐annual variation within the Nazca booby foraging area is dominated by the ENSO. ENSO warm events (El Niño events) are characterized by higher sea surface temperature and ENSO cool events (La Niña events) by lower (Fiedler, 2002; Wang & Fiedler, 2006). El Niño and La Niña conditions are associated with good and poor breeding performance, respectively, for Nazca boobies early in the breeding cycle (atypically for an eastern Pacific seabird; Tompkins & Anderson, 2021). The 5 years of our study include ample variation in sea surface temperature, including a very strong El Niño event during the 2015 breeding season and moderate/weak La Niña events during the 2011 and 2016 seasons, respectively (Figure S4). Sea surface temperature (“SST”) was averaged across the Nazca booby foraging range (a polygon covering the 95% quantile‐based interval of longitudes and latitudes calculated from the combined foraging trips of all Nazca boobies during this study) before being time‐matched (Aqua MODIS satellite, SST, 0.025 degrees, Pacific Ocean Lon ± 180; 8‐day temporal resolution) to each foraging absence (Supplementary Material). At high trophic levels, environmental influence on foraging opportunities probably integrates environmental changes across relatively large spatial scales, explaining our focus on average conditions across the area accessible to breeders from our colony.

Cloud cover affects ambient light level, which we hypothesized to be important for this plunge‐diving, visual predators that locate, and capture, prey items up to five meters beneath the water surface (Zavalaga et al., 2012). We time‐ and location‐matched values of cloud cover (from the European Centre for Medium‐Range Weather Forecasts; ECMWF) to all locations recorded during each foraging trip of Nazca boobies using the Env‐DATA annotation system in Movebank (www.movebank.org; Dodge et al., 2013). “Cloud Cover” was the proportion of each model grid cell covered by clouds within 2 km of the Earth's surface (0 = no clouds) at a 3‐h temporal and 0.75‐degree spatial resolution. We then averaged Cloud Cover (daylight only) across each foraging absence to match the temporal scale of the response variables.

The median breeding date of the population varied considerably during the 5 years of this study (from November 25 to December 14), with much of the inter‐annual variation not explained by the ENSO (Tompkins & Anderson, 2021). Population‐level shifts in breeding date (“Median Breeding Date”) probably track the quality of the environment during the breeding period (Anderson, 1989) and were included as an additional (indirect) measure of the environment (Table 1). Two‐way interactions allowed age effects to vary with each measure of environmental quality. For Stage 2 analyses, if Age appeared in the top model within Stage 1, the data were restricted to ages 5–22 because ages 4 and 23–25 were represented in only two of the five breeding seasons sampled.

To reduce model complexity, the predictors SST, Cloud Cover, and Median Breeding Date (and their interactions with Age) were evaluated in separate models. All models included crossed random intercepts for ID (accommodating repeated measurements of some individuals in different breeding seasons) and DepartGroup, and the fixed effects Date and Logger; the inclusion of Wing Loading, the environmental predictor, and the interaction terms varied across the model set. The top model (lowest AICc) best‐explained variation in the data; we also considered models falling within 2 ΔAICc of the top model (top model set) to be highly supported, unless they were more complex versions of a nested model with a lower AICc. In this scenario, the more complex model incurs a low penalty for the additional complexity (2 AICc units for each additional parameter) and thus can fall within 2 ΔAICc despite explaining little variation in the response (Burnham & Anderson, 2010). A focal predictor's effect size and the span of the 95% CI (relative to zero) of its coefficient estimate also affected inference (Arnold, 2010).

We checked for collinearity between predictors (Table S4). Absence Duration, Total Distance, and Time Searching were square‐root‐transformed, and Mass Gain/hour was log‐transformed, before analysis to satisfy the assumption of normally distributed residuals. Residuals were plotted against the fitted values and each predictor to visually assess normality and homoscedasticity and to confirm linear relationships between each predictor and the response. Final sample sizes for each trait and Sex combination are reported in Tables S6 and S7.

#### 2.4.3 Spatial segregation

To assess spatial segregation in foraging locations across AgeGroups (Young, Middle Age, Old, Oldest; see “Tagging Methods”), we first described general (95% utilization distribution kernels; UD) and core use (50% UD) areas by AgeGroup for only the locations labeled “searching” by the EMbC categorization, using the *adehabitatHR* R package (Calenge, 2006). We created UDs for each AgeGroup using the “kernelUD” function, with a grid size of 500 m and the value of the smoothing parameter *h* set to the minimum value calculated using the function “href” for each group in a given comparison (Calenge, 2006); this resulted in *h* values between 15.3 and 30.7 km. AgeGroup‐specific UDs were created from the combined tracks of all individuals within a category, and thus the resulting probability density is not equally weighted across individuals (it allows longer trips to contribute more data). A metric of similarity (Bhattacharyya's Affinity, “BA”; Fieberg & Kochanny, 2005) for each pairwise combination of AgeGroups estimated the degree of overlap. BA takes a value between 0 (indicating no overlap) and 1 (complete overlap) and has lower bias in estimates of spatial overlap than some other statistics (Fieberg & Kochanny, 2005). A randomization approach measured the statistical significance of any spatial differences between AgeGroups: all foraging tracks within a given comparison (e.g., Young vs. Middle Age males) had their AgeGroup randomly reassigned 1000 times (preserving the original proportion of tracks within each group) and a new overlap score was calculated for each iteration (to create a null distribution). The proportion of randomized overlaps (BA statistics) smaller than the observed overlap was used as a *p* value (*P*; following Breed et al., 2006). Age effects on searching locations were evaluated separately by sex, first for all data, and then separately within each Breeding Season. With four AgeGroups, six pairwise comparisons were required to assess age effects on searching location within each Sex and Breeding Season combination. To reduce Type I error from multiple comparisons, we adjusted the critical value for each test using the false discovery rate procedure (Benjamini & Hochberg, 1995), setting the false discovery rate to 0.05.

## 3 RESULTS

### 3.1 Stage 1: Age effects on foraging traits

**FIGURE 1:**
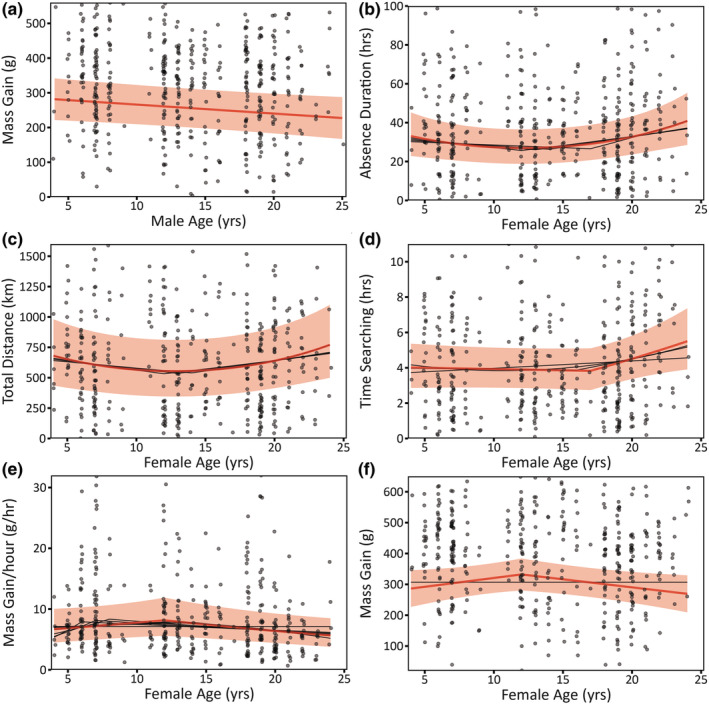
Age effects on foraging traits are strongly supported for male (a) Mass Gain, and female (b) Absence Duration, (c) Total Distance, (d) Time Searching, (e) Mass Gain/hour, and (f) Mass Gain. Red lines are model predictions from the top model from Stage 1; red‐shaded regions are 95% CIs. Black lines are model predictions from the other top models in the top model set, illustrating model selection uncertainty in the Age function's form. We modeled sqrt(Total Distance), sqrt(Absence Duration), sqrt(Time Searching), and log(Mass Gain/hour), but back‐transformed the predicted values to show effect size. Points show the raw data; darker shades indicate overlap (jittered horizontally).

For male Nazca boobies, age effects on foraging traits were supported strongly only for Mass Gain, considering AICc comparisons with the null (no age) model and whether the 95% CIs on the coefficients describing the age function included zero (Table S6). Older males gained less mass after foraging than younger birds did, a linear decline with age (Age: β = −2.58 [95% CI: −4.61, −0.36]; Figure 1a). For male Mass Gain/hour (the rate of mass gain), a linear age effect received limited support, despite appearing in the top model, because the model without age performed nearly as well (∆AICc = 1.88; Table S6) and the 95% CI on the Age coefficient included zero (Age: β = −0.006 [95% CI: −0.013, 0.000]; Figure S5). The null (no age) model was the top model for male Absence Duration, Total Distance, and Time Searching.

For females, age appeared in the top model for every foraging trait. Age effects on foraging traits were supported strongly for Absence Duration, Total Distance, and Time Searching, with the top model for each trait outperforming the null model by ∆AICc > 3 (Table S7). In our cross‐sectional sample, Absence Duration and Total Distance first decreased, and then increased, with age (Figure 1b,c). Some model selection uncertainty affected the form of the age function (e.g., threshold vs. quadratic; Figures 1b,c; Table S7), but not this conclusion. All age coefficients for Total Distance were distinct from zero (Age: β = −0.85 [95% CI: −1.50, −0.18]; Age^2^ β = 0.03 [95% CI: 0.01, 0.06]; Figure 1c). The linear age coefficient also excluded zero for Absence Duration (Age: β = −0.21 [95% CI: −0.38, −0.03]), although the quadratic coefficient (narrowly) did not (Age^2^: β = 0.01 [95% CI: 0.00, 0.01]; Figure 1b). Age effects on Time Searching were also highly supported: birds older than age 17 increased their time spent actively searching compared to young and middle‐aged birds (a threshold function; Age ≤ 17: β = −0.01 [95% CI: −0.02, 0.01]; Age > 17: β = 0.06 [95% CI: 0.01, 0.10]; Figure 1d).

Although age effects also appeared in the top model for female Mass Gain/hour and Mass Gain, the null (no age) model appeared within the top model set (∆AICc of 1.59 and 0.59, respectively; Table S7). Age‐related changes in performance were largely restricted to late life for these two variables, explaining the model selection uncertainty regarding the importance of age. Mass Gain/hour was relatively stable through age 12 (Age ≤ 12: β = 0.011 [95% CI: −0.004, 0.025]; following a single‐threshold function), and then declined (Age > 12: β = −0.013 [95% CI: −0.027, −0.002]; Figure 1e). Patterns were similar for Mass Gain: Mass Gain was relatively stable (slightly increasing) through age 12 (Age ≤ 12: β = 5.71 [95% CI: −0.91, 12.34]; following a single‐threshold function), and then declined (Age > 12: β = −5.23 [95% CI: −0.40, −10.05]; Figure 1f).

Comparing the two sexes, age effects were strongly supported in males for Mass Gain, and in females for Mass Gain/hour, Mass Gain, Absence Duration, Total Distance, and Time Searching, implying an Age by Sex interaction for foraging performance, with typically stronger aging in females. In Stage 1, we evaluated the sexes in separate models to allow the selection of different age functions in each sex (following previous results for breeding traits; Tompkins & Anderson, 2019); this did not permit an explicit test of an Age by Sex interaction. Post hoc, we combined the male and female data for each trait and added an Age by Sex interaction to the quadratic age function model from Stage 1 (linear + quadratic Age effects, each interacting with Sex). The interaction coefficients, and the span of their 95% CIs, were used to directly evaluate support for stronger female aging. Many interaction coefficient estimates were in the expected direction (stronger female aging for Absence Duration, Total Distance, and Time Searching); however, the 95% CIs included zero, sometimes narrowly, in each case (Table S8). Thus, sex differences in aging for foraging performance may exist in this system, but the support is equivocal.

### 3.2 Stage 2: Environmental effects on foraging traits

The warmest sea surface temperatures during the study were recorded during the 2015–2016 El Niño event (Figure S4). SST appeared in the top model for each sex/response variable except male Mass Gain (Table 2). For males and females, foraging traits suggested favorable conditions when SST was relatively warm: high Mass Gains/hour was achieved through short Absence Durations, Total Distances, and Time Searching (coefficients in Table S9); low Mass Gains (in females only) did not outweigh the positive effects of short trips on Mass Gain/hour. In males, Mass Gain followed shifts in the median population breeding date: in seasons with relatively late laying, males gained less mass while foraging.

**TABLE 2:** Models explaining variation in foraging traits from Stage 2 analyses of environmental predictors.

	Males						Females					
**Response variable**	**Age effect**	**Other fixed effects**	***k***	**ΔAICc**	***ω***_***i***_	***R*** ^**2**^_**m**_ **/*R*** ^**2**^_**c**_	**Age effect**	**Other fixed effects**	***k***	**ΔAICc**	***ω***_***i***_	***R***_**m**_^**2**^ **/*R***_**c**_^**2**^
**Mass Gain/** **hour**	**Age**	**SST**	**8**	**0.00**	**0.51**	**0.13/0.34**	**One T (12)**	WL + Age*SST + (Age – T_**1**_ **)**_**+**_ *SST	**12**	**0.00**	**0.98**	**0.16/0.32**
	Age	WL + SST	9	1.94	0.19	0.13/0.35						
**Mass Gain**	**Age**	**WL + MBD**	**9**	**0.00**	**0.52**	**0.20/0.29**	**One T (12)**	**WL + SST**	**10**	**0.00**	**0.48**	**0.18/0.25**
	Age	WL + Age*MBD	10	1.03	0.31	0.20/0.29	One T (12)	WL + Age*SST + Age^2^*SST	12	0.38	0.40	0.18/0.25
**Absence Duration**		**WL + SST**	**8**	**0.00**	**0.99**	**0.14/0.31**	**Age + Age** ^**2**^	**WL + SST**	**10**	**0.00**	**0.66**	**0.21/0.33**
							Age + Age^2^	WL + Age*SST + Age^2^*SST	12	1.33	0.34	0.21/0.34
**Total Distance**		**WL + SST**	**8**	**0.00**	**0.98**	**0.17/0.44**	**Age + Age** ^**2**^	**WL + SST**	**10**	**0.00**	**0.85**	**0.21/0.49**
**Time Searching**		**WL + SST**	**8**	**0.00**	**0.83**	**0.15/0.38**	**One T (17)**	**WL + SST**	**10**	**0.00**	**0.60**	**0.24/0.32**

*Note*: Top models (in bold) are within ΔAICc of 2 and are not a more complex version of a simpler, nested model; models within 2 ΔAICc are shown, Tables S10 and S11 contain complete model rankings. The number of parameters (k), AICc difference from the top model (ΔAICc), and Akaike weights (*ω*_*i*_) are reported. Conditional *R* ^2^ (*R* ^2^_c_) includes, and marginal *R* ^2^ (*R* ^2^_m_) excludes, variance explained by DepartGroup and ID (random effects in all models) and are reported for the top model. Date and Logger appeared as fixed effects in all models. Cloud = Cloud Cover; WL = Wing Loading, MBD = Median Breeding Date. Main effects with interactions are simplified (e.g., Age + SST + Age⁎SST is written as “Age⁎SST”). “One T (age)” denotes a single‐threshold function.

Age‐related changes in female Mass Gain/hour depended on sea surface temperature: during years with cold SSTs (relatively “poor” environments during incubation, as during La Niña), Mass Gain/hour declined between middle age and old age (consistent with senescence), but as sea surface temperature increased, this age‐related decline was reduced or even reversed (in the warmest temperatures; Figure 3). The pattern thus follows prediction 3: age effects were attenuated in good‐quality environments. The interaction slope that describes the change in aging rate for a one‐unit increase of SST was distinct from zero only for the second part of the threshold age function (for Age > 12: interaction slope = 0.009 [95% CI: 0.001, 0.017]; Table S9), suggesting that increases in performance between young and middle‐aged birds are relatively constant across environmental variation.

### 3.3 Spatial segregation

**FIGURE 2:**
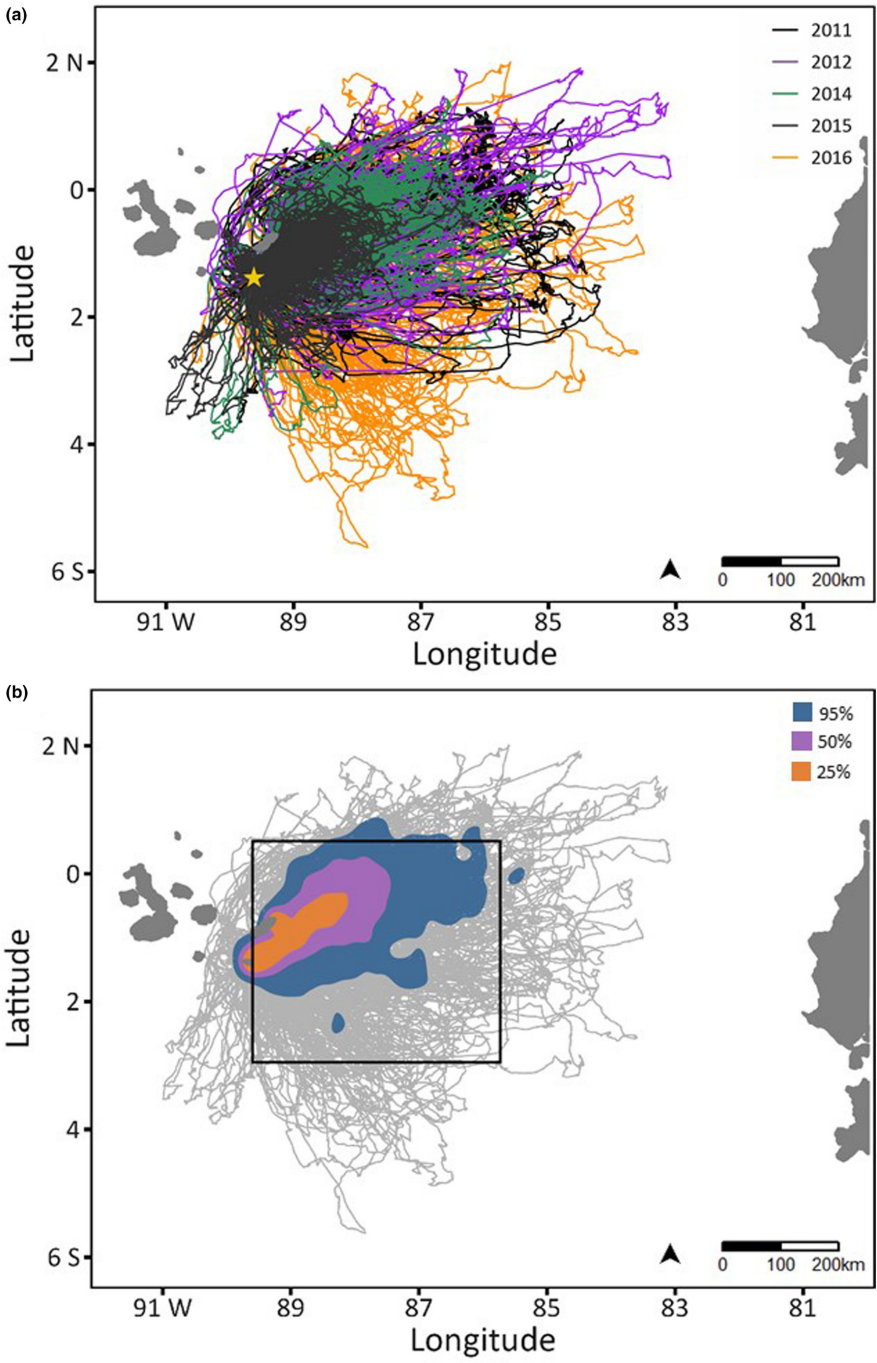
Foraging routes for Nazca boobies during egg incubation from the breeding colony at Punta Cevallos, Isla Española, shown by a yellow star at 1°23′ S, 89°37′ W. (a) Routes colored by Breeding Season (see Figure S6 for a multipanel display). (b) Kernel densities (see legend) of all locations, regardless of activity. The black box in B bounds the area where environmental variables were measured (Supplementary Material).

Foraging Nazca boobies frequently occupied a corridor northeast of the breeding colony, with most routes confined to the eastern wedge of ocean surrounding the colony (Figure 2). Nazca boobies consistently depart the colony flying in a northeast direction (see table S6 in Howard et al., 2021), explaining the concentrated use of the corridor just northeast of the colony, followed by highly variable use of space thereafter. Areas used for localized searching by males and females overlapped substantially (pooled across years, observed BA for 95% UD = 0.89), but less than expected by chance (*p* < 0.001; Table S12). Thus, we analyzed age effects on searching locations separately by Sex and Breeding Season, acknowledging differences in total trip distance and spatial coverage across years (Figure 2a, Figure S6, Table S13).

Spatial segregation among the AgeGroups was evident in 2016, but not in the other years; in most instances, Old (ages 17–20) and Oldest birds (ages 21–25) were distinguished spatially from Young birds (ages 5–9; Table S14). Absence Durations were shortest during the strong El Niño of 2015 and longest during the weak La Niña of 2016 (see figure 1 in Howard et al., 2021 and table S35 in Howard, 2021); here, we focus on the detailed patterns from these two extremes (Figure 4, Figure S6; see full results in Tables S14). In 2015, spatial overlap of the four AgeGroups was similar to that expected by chance (Table S14), consistent with the small area used for searching (Figure 4). In contrast, in 2016 Young males and Young females occupied somewhat spatially distinct general use areas (95% UDs) from the Oldest birds. Core use areas (50% UDs) of Young birds were also somewhat distinct from those of Middle Age (males) and Old/Oldest birds (females; Figure 4, Table S14). In 2016, Young birds searched to the southeast of the colony more than other AgeGroups.

## 4 DISCUSSION

We predicted lower foraging performance in young and old incubating Nazca boobies than for middle‐aged ones. Age was expected to interact with environmental quality, either because favorable environments reduce age‐related discrepancies in performance (prediction 3), or because favorable environments enhance age affects (e.g., if middle‐aged birds are better able, or more motivated, to take advantage of resource abundance than other age classes are; prediction 4). Most foraging traits responded to variation in SST associated with the El Niño‐Southern Oscillation, but age largely did not affect birds' response to environmental variation. Under relatively warm SSTs (as during El Niño), all age classes reduced foraging trip length (Absence Duration, Total Distance) and time spent actively foraging (Time Searching), resulting in a higher foraging efficiency (Mass Gain/hour) despite accruing a lower Mass Gain (females only) than under relatively cool SSTs. These foraging results from incubating Nazca boobies match ENSO influences on breeding traits expressed early in the season (Tompkins & Anderson, 2021), linking improved food availability during El Niño‐like conditions to large clutch sizes (all ages) and high breeding probabilities and early laying (in young birds).

Only female Mass Gain/hour (rate of mass gain) varied with an age by SST interaction: foraging efficiency declined after age 12 during all but the most favorable (warmest sea surface) environments. Age effects shrank when the environment was relatively benign (warm SST was associated with high rates of mass gain and short trips), suggesting that old birds may be able to overcome any physiological deficits associated with senescence when resources are abundant. Age, while only interacting with SST for female Mass Gain/hour, also affected other aspects of foraging ability in our cross‐sectional sample. Reduced foraging efficiency (and its components; Mass Gain, Absence Duration, Total Distance, and Time Searching) in old females suggested deficits in resource acquisition (this study) as explanations for previous results showing poor late‐life breeding performance and survival in this species, particularly for females (Tompkins & Anderson, 2019). In males, only Mass Gain changed with age, declining slowly with age.

### 4.1 Age effects on foraging traits

This study revealed lower Mass Gain/hour (rate of mass gain) of female Nazca boobies during the incubation period during all but the warmest (most favorable) sea surface temperature environments. Explaining age effects on Mass Gain/hour, old females traveled longer distances, took longer absences, and spent more time searching than middle‐aged females did (e.g., differences of 230 km, 15, and 2 h, respectively, between the ages of 12 and 24), yet still returned to the nest with slightly lower mass gains (e.g., a reduction of 60 g from age 12 to 24). During incubation, foraging breeders dictate the shift length and fasting duration of their mate. Thus, longer absences of old females impose a cost on the nest‐bound partner, increasing the probability that he will abandon their clutch (42% of nest failures happen during incubation in this population; DJ Anderson, unpub. data). Despite this risk, old females may prioritize self‐feeding, to restore body reserves, over their clutch's survival, as predicted by life history theory (Stearns, 1992) for a long‐lived species at one end of the axis of trade‐off between self‐maintenance and reproductive investment. How the partners of these old females respond to relatively long incubation shifts remains unknown; our sampling strategy did not target mated pairs, and age effects on male Absence Duration are uninformative because of frequent mate rotation (Maness & Anderson, 2007, 2008). Females often divorce their mate to pair with a recent non‐breeder (Maness & Anderson, 2008), disrupting the correlation between male and female ages (Tompkins & Anderson, 2019). Males paired with old females may lengthen their own trips to recover (maintaining coordination, but with both sexes suffering long shifts at the nest), or take on a larger burden of incubation if coordination between pair members breaks down (Patrick et al., 2020).

In contrast, evidence of age‐related changes in male foraging performance was much weaker than for females. Males showed no change in Absence Duration, Total Distance, or Time Searching with age. Although male Mass Gain declined slowly across the lifespan (a 19% reduction from the youngest to the oldest ages; Figure 1a), Absence Duration did not, so reductions in Mass Gain were not strong enough to reduce Mass Gain/hour substantially in old age (although Age appeared in the top model for Mass Gain/hour, the coefficient estimate wasn't distinct from zero). These results imply an Age by Sex interaction, with stronger female aging for all characteristics except Mass Gain, but the inference from an explicit test is weak: the coefficients are in the expected direction, but the 95% CIs include zero (see Results). While we fall short of providing strong support for stronger female aging in foraging traits in the current dataset, this remains an interesting hypothesis to revisit in future studies, potentially with respect to diving characteristics or diet—factors that may be shaped by the sexual size dimorphism in this species.

During poor food years, female offspring reach independence at poorer body condition than male offspring, leading to poorer sub‐adult survival (Maness & Anderson, 2013) and finally to under‐representation of females in the adult population: 60% of adults are male (Maness & Anderson, 2007). Males are regularly rotated in and out of the breeding population by female choice (Maness & Anderson, 2007, 2008), homogenizing the schedules of male reproductive effort at a lower level than those of females (Maness & Anderson, 2007). Females recruit into the breeding population at a younger age than males do (Champagnon et al., 2018; Maness & Anderson, 2013), further accelerating females' accumulation of reproductive costs compared to males. Then, in old age, females experience earlier and/or stronger actuarial and reproductive senescence than males (Tompkins & Anderson, 2019). Thus, female‐biased senescence in Nazca boobies may be explained as a long‐term cost of reproduction (Bonduriansky et al., 2008; Promislow, 2003; Williams, 1957). However, the expected early‐late life fitness trade‐offs were not recovered in an observational study (Tompkins & Anderson, 2019), illustrating the challenge of measuring reproductive investment in the wild.

Independent of any sex differences in old‐age physiological decline, the breeding/not‐breeding filter limiting participation in this study to breeders may contribute to female‐biased senescence in foraging traits in this subset. Females are the limiting sex and retain a high breeding probability into old age, while that of old males falls precipitously (Tompkins & Anderson, 2021). The subset of old males that enter the breeding pool may be selected by females (females control mate choice in this species; Maness & Anderson, 2008) because they are in relatively good condition for their age. This proposed process is akin to that of selective disappearance (low‐quality phenotypes die early), with age at death (not breeding participation) filtering the phenotypes sampled in old age classes. Mate choice dynamics that exclude senescent males from the breeding pool may be more likely than selective disappearance to conceal age effects on foraging because old males breed infrequently (Tompkins & Anderson, 2019) and longitudinal analyses using decades of breeding records have found little scope for selective disappearance to bias estimated age effects (see figure S8 in Tompkins & Anderson, 2019, 2021). Capturing the full range of age‐dependent changes in food acquisition may require new insights into the condition and foraging abilities of non‐breeders (pre‐breeders and skippers), particularly for males.

Turning to young adults, early‐life improvement in foraging performance was absent (males), or relatively subtle (females), compared to that seen for reproductive traits. Limited periods of steep improvement (through age 6–10, depending on the trait) are followed by a plateau in middle age for reproductive traits (Tompkins & Anderson, 2019, 2021), in contrast to the slow and extended improvements in female foraging implied by the best‐performing quadratic age functions for Total Distance and Absence Duration and by the marginally‐supported linear increases in Mass Gain/hour and Mass Gain (through age 12; from a single‐threshold function) and Time Searching (through age 17). Although naïve seabirds improve in foraging efficiency (Fayet et al., 2015) and body condition (Weimerskirch, 1992) during an extended sub‐adult phase, recruiting to the breeding population poses new challenges. Accordingly, young and/or inexperienced parents are less efficient foragers (Daunt et al., 2007; Galbraith et al., 1999), showing deficits in prey delivery (Limmer & Becker, 2009; Navarro et al., 2010), trip duration (Frankish et al., 2020), and diving (Cunningham et al., 2017; Le Vaillant et al., 2013; Lescroël et al., 2023). Nazca boobies show only weak evidence of reduced foraging performance early in their breeding years, suggesting that recruitment in this species demands a high level of foraging competence (particularly for males) and implicates other factors (e.g., poor coordination between pair members, a lack of breeding experience, and/or deferred investment toward reproduction) in the poor breeding success of young boobies (Forslund & Pärt, 1995; Patrick et al., 2020). Consistent with our results, young breeders have similar body masses to those of middle‐aged breeders when they depart for, and return from, foraging absences (Howard et al., 2021; Table S15). As in albatrosses (e.g., Weimerskirch, 1992) and gulls (e.g., Pyle et al., 2001), delaying the first breeding until foraging proficiency and body condition are sufficient for the current environmental conditions may protect survival (and future breeding opportunities) during the first breeding attempt (Ricklefs, 1983).

### 4.2 Environmental effects on foraging

**FIGURE 3:**
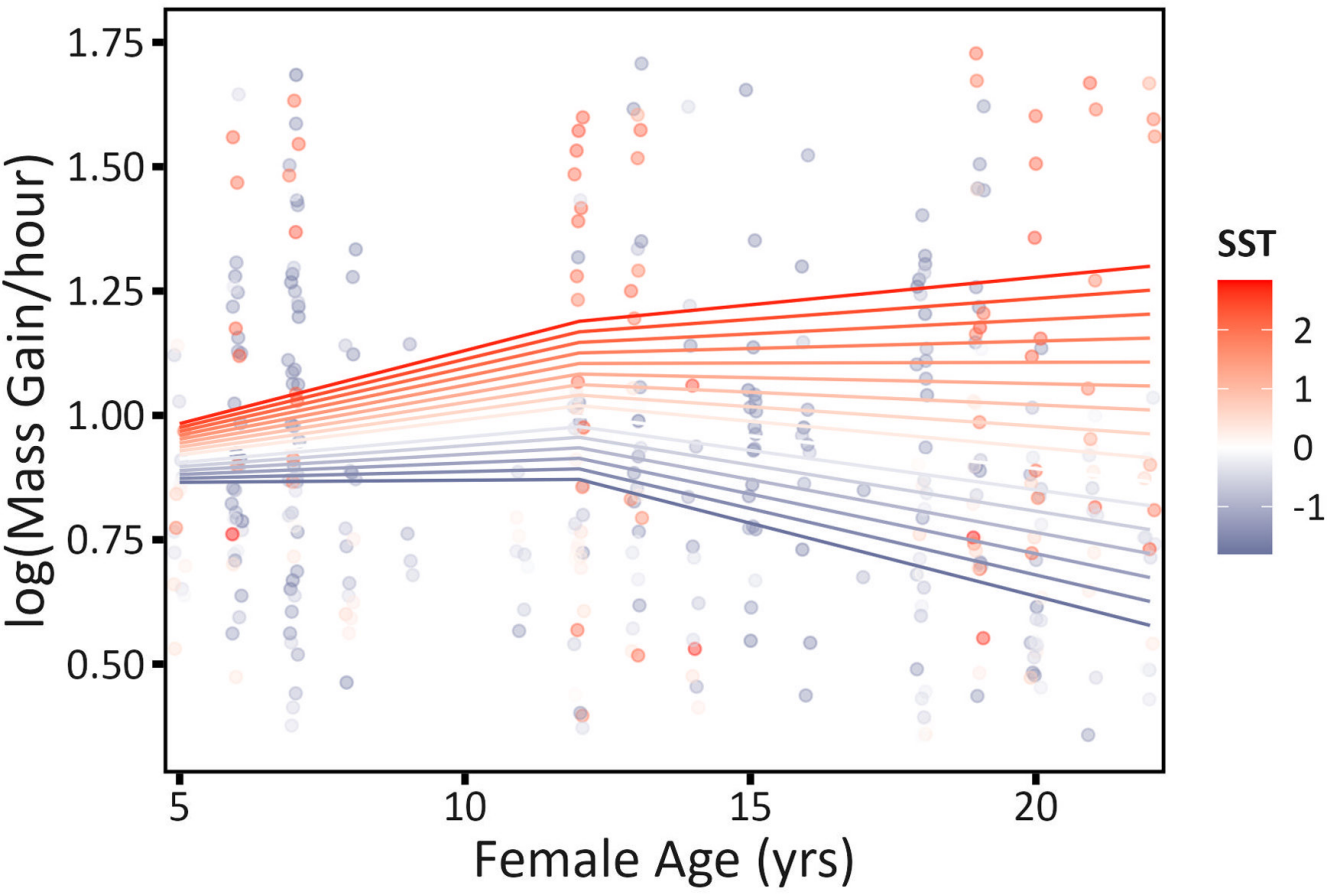
Interactive effects of Age and Sea Surface Temperature (SST) on female Mass Gain/hour. Lines show model‐predicted aging patterns across the range of SST observed in our 5‐year study and were calculated holding continuous predictors (other than age) at their mean value and factor Logger at i‐gotU® GT‐120. Points show raw data (with color corresponding to the SST during each foraging trip).

Mixed evidence for senescence in seabird foraging performance (often from the same species or population) has been attributed to an age‐by‐environment interaction (among other factors; e.g., Catry et al., 2006, Frankish et al., 2020, Lecomte et al., 2010). The current study covered five breeding seasons, including striking variation in SST within the Nazca booby foraging range (Figure S4), and revealed an interaction between age and SST affecting female Mass Gain/hour (Figure 3). When sea surface temperatures were relatively cool (a poor environment characterized by long foraging trips and more time searching), old females had lower foraging efficiency than middle‐aged ones. But apparent late‐life declines in foraging efficiency disappeared as the quality of the environment increased (as SST warmed), emphasizing the difficulty of detecting senescence in foraging performance in the wild. Environments associated with relaxed foraging requirements were expected to shrink age‐related differences in foraging performance—the pattern observed for female Mass Gain/hour—and such an environment may have allowed old birds to overcome intrinsic performance deficits due to physiological decline and/or low motivation (Laaksonen et al., 2002; Ratcliffe et al., 1998; Sydeman et al., 1991). Consistent with the pattern shown for female Mass Gain/hour, late‐life reductions in fledging success in female Nazca boobies start at an older age when environmental quality is relatively high (Tompkins & Anderson, 2021), broadly consistent with the pattern observed in female Mass Gain/hour (stronger aging in a poor environment), and confirming that age‐by‐environment interactions can be an important source of variation in performance in this species. Age‐by‐environment interactions were not supported for the other foraging traits examined: Mass Gain, Absence Duration, Total Distance, and Time Searching.

**FIGURE 4:**
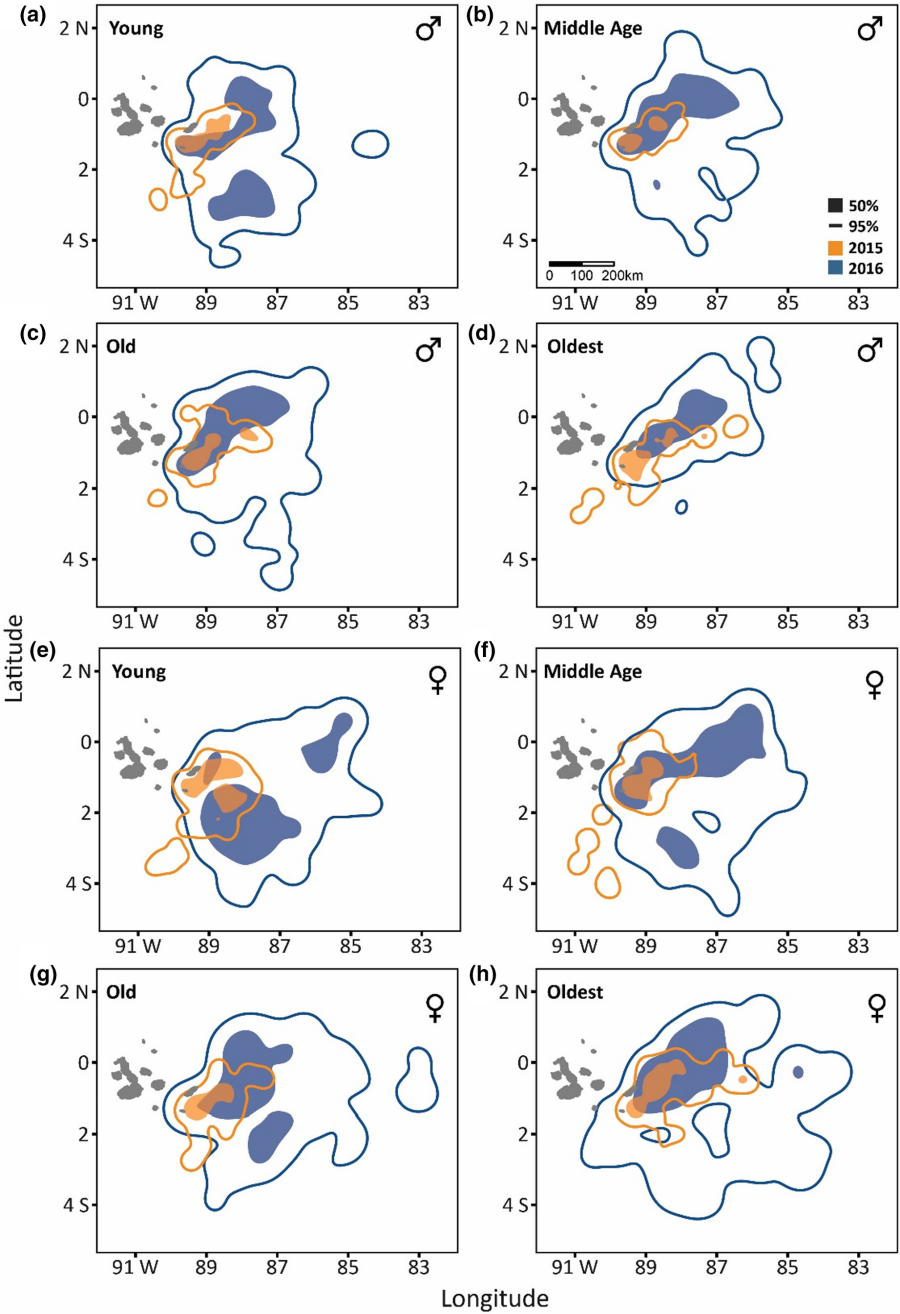
For male (a–d) and female (e–h) Nazca boobies, foraging locations of Young (a), Middle Age (b), Old (c), and Oldest (d) incubators from the Punta Cevallos colony during the 2015 El Niño (orange) and the 2016 La Niña (blue). Solid lines indicate general use (95%) and filled polygons indicate core use (50%) areas.

Patterns of spatial overlap varied by age and breeding season, also suggesting an age‐by‐environment interaction. The four AgeGroups showed the lowest overlap of localized searching areas in the 2016 weak La Niña. Reduced spatial overlap in 2016 may signal competitive exclusion of, or avoidance by, Young Nazca boobies from key foraging areas, as proposed for young gulls (Navarro et al., 2010) and old albatrosses (Frankish et al., 2020), or may reflect young birds' lower efficiency at locating profitable foraging patches in a poor environment. To translate age‐ and environment‐related patterns of spatial occupancy to consequences for reproductive performance, young birds should pay a cost to the quality of food acquired and/or its rate of delivery for performing local searches where other age classes do not. This cost was not paid in terms of Mass Gain/hour or its components, but diet composition and quality were not evaluated here and may show the expected age‐by‐environment interaction (e.g., Limmer & Becker, 2009), building on slight age‐related increases in Absence Duration, Total Distance, Time Searching, and Mass Gain/hour (females) to help explain middle‐age superiority in breeding performance.

Relatively warm sea surface temperatures are associated with high foraging performance in male and female Nazca boobies (this study, Howard et al., 2021), reducing Absence Duration, Total Distance, and Time Searching (Table S9) and resulting in higher Mass Gain/hour. Temporal variability in the Eastern Tropical Pacific is dominated by the ENSO, shifting between warming (El Niño) and cooling (La Niña) phases (Fiedler, 2002). With seasonal increases in ocean temperature controlled (see Supplementary Material), differences among breeding seasons explained most of the variation in our environmental variable SST (*r* ^2^ = .80; Figure S3, Table S5), suggesting that the observed temporal variation in foraging traits follows inter‐annual changes in the ENSO (Howard et al., 2021). Breeders confront a trade‐off during the incubation period between self‐feeding and survival of the clutch: parents must coordinate their reciprocal absences well to avoid their nest‐bound partner abandoning the nest. Contrasting with predictions based solely on primary productivity (El Niño bad, La Niña good) and results from other seabirds (e.g., Ancona et al., 2011, 2012), for Nazca boobies warm SST during incubation marks environmental conditions in which the trade‐off is easier to manage. Warm SST may positively influence the availability and abundance of prey, resulting in increased flying fish availability (as recorded from the Gulf of Mexico; Churnside et al., 2017), an important prey item for Nazca boobies during the study period (Tompkins et al., 2017). Thus, effects of SST on the distance and duration of a foraging absence (this study; Howard et al., 2021) inform improved breeding probability (for young birds) and clutch size in El Niño‐like conditions (Tompkins & Anderson, 2021). Here we measured Nazca booby foraging performance during incubation, but foraging conditions during El Niño are predicted to change from resource‐rich to resource‐poor as an El Niño event unfolds across the 7–8 months of a breeding season. Offspring survival is negatively associated with each ENSO extreme (Anderson, 1989; Champagnon et al., 2018; Tompkins et al., 2017; Tompkins & Anderson, 2021), and parallel foraging analyses during chick‐rearing are required to fully understand the links between oceanographic changes, food availability, and reproductive success across the entire breeding period.

## 5 CONCLUSIONS

We used high‐quality GPS data spanning 5 years to address the prediction that an individual's age conditions its foraging performance under environmental variation. Early‐life improvements in foraging ability are well documented in seabirds (e.g., Daunt et al., 2007; Frankish et al., 2020; Galbraith et al., 1999; Lescroël et al., 2019, 2023; Limmer & Becker, 2009) but evidence of physiological senescence degrading late‐life foraging performance has been mixed (observed in Catry et al., 2006, Frankish et al., 2020, Galbraith et al., 1999, but not in Elliott et al., 2015, Froy et al., 2015, Lescroël et al., 2019). Senescence in foraging ability may be revealed only under a challenging environment, a pattern observed for Mass Gain/hour in female Nazca boobies. Somewhat stronger evidence of late‐life declines in female (vs. male) foraging performance aligns with earlier and/or steeper reproductive senescence in female fledging success and survival compared to those of males (Tompkins & Anderson, 2021) and with earlier reductions in the bite force of old females (Rebol & Anderson, 2022). Taken together, these results paint a picture of enhanced physiological senescence in older female Nazca boobies. Mate‐choice dynamics may also restrict the breeding participation (thus, study participation) of old males in poor condition, further contributing to the detected female bias in age effects. An earlier two‐year study failed to detect extended Absence Durations and reduced Mass Gain/hour in old age (Total Distance and Time Searching were not examined; Howard et al., 2021), emphasizing the difficulty of detecting relatively subtle age‐related changes for traits that are highly responsive to environmental change across a range of spatial and temporal scales. Modeling the sexes separately, and allowing them to have different optimal age functions, was key to that effort here. Besides being of interest in its own right, carefully considering sex in the context of physiology, behavior, life history, and demography may be important to detecting senescence in foraging traits, even within (serially) monogamous species practicing biparental care.

Most studies of age‐related variation in foraging in seabirds occur during the chick‐rearing phase (Catry et al., 2006; Cunningham et al., 2017; Elliott et al., 2015; Frankish et al., 2020). By studying parents during the incubation period, the dataset reduces the possibility of selective sampling inherent to studies during the chick‐rearing period: high‐quality individuals that did not fail during incubation may be over‐represented during chick‐rearing, constraining the range of response variables and minimizing the true effects of age on performance. Our results show that young and old female boobies take longer foraging absences during incubation than middle‐aged birds, traveling a greater distance, and suffering a lower foraging efficiency (Mass Gain/hour) as a result. Thus, age‐related changes in foraging performance start during incubation and probably accumulate across the entire breeding season, and could contribute to observed reproductive senescence (Tompkins & Anderson, 2019).

## AUTHOR CONTRIBUTIONS

**Jennifer L. McKee:** Conceptualization (lead); data curation (lead); formal analysis (lead); funding acquisition (supporting); investigation (equal); methodology (lead); project administration (equal); supervision (equal); validation (lead); visualization (lead); writing – original draft (lead); writing – review and editing (equal). **Emily M. Tompkins:** Conceptualization (supporting); data curation (lead); formal analysis (supporting); investigation (supporting); methodology (supporting); supervision (equal); validation (equal); writing – review and editing (equal). **Felipe A. Estela:** Conceptualization (supporting); data curation (supporting); investigation (supporting); methodology (supporting). **David J. Anderson:** Conceptualization (equal); funding acquisition (lead); methodology (supporting); project administration (lead); resources (lead); supervision (equal); writing – review and editing (equal).

## CONFLICT OF INTEREST STATEMENT

The authors declare no conflict of interest.

## Supporting information


Data S1.

## Data Availability

Data files are available from the WakeSpace database: https://wakespace.lib.wfu.edu/handle/10339/102095.
