# Supplementary material for: Age effects on Nazca booby foraging performance are largely constant across variation in the marine environment: Results from a 5‐year study in Galápagos
Source: Ecol Evol. 2023 Jun 9;13(6):e10138. doi: 10.1002/ece3.10138 (PMC10253949; doi:10.1002/ece3.10138)
Supplement: Supplementary file 1 — Data S1. [file ECE3-13-e10138-s001.docx]

# SUPPLEMENTARY MATERIAL

## 1. Data Processing

Bird capture and logger deployment lasted <8 minutes and birds renewed normal activity soon after release. A minority of tagged birds (2.4%) departed the nest before their mate returned (i.e., they abandoned the clutch before the mate returned from a long foraging trip). These birds took longer Absence Durations than those that did not abandon (Wilcoxon rank sum test, Z = -5.61, p <0.01), and we excluded their data (36 absences) from our analyses. Incomplete foraging absences (e.g., the logger turned off before the bird’s return) were also removed (9.9% of remaining data). Lastly, foraging absences with >30% of the daylight GPS locations missing (27 absences, due to intermittent GPS logger failures; see below) were excluded from response variables Total Distance and Time Searching, the variables most likely to be affected by data gaps.

For three foraging variables (Absence Duration, Total Distance, and Time Searching), we focused only on the daylight period because Nazca boobies are visual predators and forage during the day; at night, they rest on the surface of the water if they have not returned to land (Zavalaga et al. 2012). These three variables are measures of foraging activity, and foraging activity happens only during the day. Dive data (1-second resolution) collected using accelerometers in 2015 and 2016 show that Nazca boobies dive for prey almost exclusively during daylight periods: only one out of 16,553 recorded dives of >0.5 m depth occurred at night; (methods described in Howard 2021; results in Figure S1). Therefore, we focused our response variables (Absence Duration, Total Distance, and Time Searching) on the daylight period. Daylight periods spanned nautical dawn to nautical dusk (when the solar angle is 12° below the horizon) and were calculated using the “sunriset” function in the R package *maptools* (Bivand and Lewin-Koh 2021); duration of daylight periods at this equatorial location had the range 13.62–14.09 hrs). Gaps in data, particularly during the daylight period, could affect the accuracy of our estimates of foraging traits because sampling frequency influences apparent distance and speed (Noonan et al. 2019). Gaps in data occurred when the GPS logger turned off spontaneously and did not record locations. We calculated the lengths of gaps during the daylight period to obtain a percentage of daylight location data missing from individual foraging absences (sum of all gaps/total daylight hours, calculated for each foraging absence). The median percentage of missing locations was 0.0%. Foraging absences missing >30% of daylight locations were removed from analyses of response variables Total Distance and Time Searching (3.5% of cases were removed). These 27 absences (with > 30% of daylight GPS positions missing) occurred at a similarly-low frequency in the two sexes (2.6% of male trips and 4.4% of female trips) and across age classes (4.2% of trips in Young birds, 1.7% in Middle Age birds; 3.2% in Old birds; 0.0% in Oldest birds). Trips with a > 30% missing data were also not a biased subset with respect to trip length (the median trip lengths for trips with < 30% missing data versus that from trips with > 30% missing data are 34 hours and 37 hours, respectively). We retained foraging absences with <30% missing daylight locations in our final analyses to balance the benefits of retaining most foraging absences collected (in terms of sample size and preserving the sampling structure across age groups) with the potential negative effects on inference from including biased estimates of Total Distance and Time Searching (which are expected to be underestimated when data are missing). Re-running Stage 1 and Stage 2 analyses using more stringent criteria (retaining absences with <20% or <10% missing information) did not affect our results (data not shown). A minority of the cases of mass gain were negative (23 birds lost mass during the foraging absence); these were removed before analyzing male and female Mass Gain/hr because this response variable was log transformed before analysis.

Explanatory variable Wing Loading was calculated for each bird, at each departure, as the ratio body weight at departure/wing area. Wing chord, but not wing area, was measured for birds in the current study. To calculate wing area from wing chord, we used a dataset from 2018 containing wing areas (m^2^) and wing chords (m) measured from 50 Nazca boobies (25 of each sex) to estimate the relationship between these two variables. The resulting linear regression allowed us to estimate wing area as: square root of wing area = 0.04+0.95*wing chord; r^2^ = 0.49). In the 50-bird dataset, wing area (m^2^) was calculated as twice the area of the outstretched wing from the bird’s midline to the wing tip, using Image J v.1.8.0 software applied to a digital photograph of the outstretched right wing taken from above, and perpendicular to, the wing surface. Wing chord length was measured as the flattened wrist-to-tip distance (m).

**Table S1.** Distribution of GPS loggers deployed on Nazca boobies by Breeding Season, logger model, Sex, and AgeGroup. Sample sizes are presented as the total number of deployments (one per bird per breeding season). Ages 15–16 were sampled in 2011–2012 only, as Middle Age birds; in later years, Middle Age was restricted to 11–14 year-olds to more conservatively isolate prime-age performance within this category.

| **Breeding Season** | **GPS Logger Model** | **Sex** | **AgeGroup** | | | |  |
| --- | --- | --- | --- | --- | --- | --- | --- |
|  |  |  | **Young**  **(4–9 yrs)** | **Middle Age (11–16 yrs)** | **Old (17–20 yrs)** | **Oldest**  **(21–25 yrs)** | **Row Total**  **(All ages)** |
|  | GT-120 | Male | 65 | 60 | 53 | 3 | 181 |
| 2011 |  | Female | 57 | 53 | 48 | 4 | 162 |
|  |  | **Total** | **122** | **113** | **101** | **7** | **343** |
|  | GT-120 | Male | 34 | 30 | 32 | 1 | 97 |
| 2012 |  | Female | 37 | 22 | 34 | 0 | 93 |
|  |  | **Total** | **71** | **52** | **66** | **1** | **190** |
|  | GT-120 | Male | 12 | 12 | 12 | 7 | 43 |
|  |  | Female | 10 | 10 | 10 | 4 | 34 |
| 2014 | GT-600 | Male | 9 | 9 | 9 | 3 | 30 |
|  |  | Female | 13 | 13 | 13 | 8 | 47 |
|  |  | **Total** | **44** | **44** | **44** | **22** | **154** |
|  | GT-120 | Male | 14 | 14 | 14 | 11 | 53 |
|  |  | Female | 7 | 7 | 7 | 7 | 28 |
| 2015 | GT-600 | Male | 16 | 17 | 18 | 11 | 62 |
|  |  | Female | 22 | 23 | 22 | 16 | 83 |
|  |  | **Total** | **59** | **61** | **61** | **45** | **226** |
|  | GT-120 | Male | 19 | 29 | 17 | 9 | 74 |
|  |  | Female | 12 | 11 | 10 | 8 | 41 |
| 2016 | GT-600 | Male | 13 | 28 | 15 | 11 | 67 |
|  |  | Female | 20 | 27 | 20 | 17 | 84 |
|  |  | **Total** | **64** | **95** | **62** | **45** | **266** |
|  |  | **Male** | 182 | 199 | 170 | 56 | 607 |
| **All** | **All** | **Female** | 178 | 166 | 164 | 64 | 572 |
|  |  | **Total** | **360** | **365** | **334** | **120** | **1,179** |


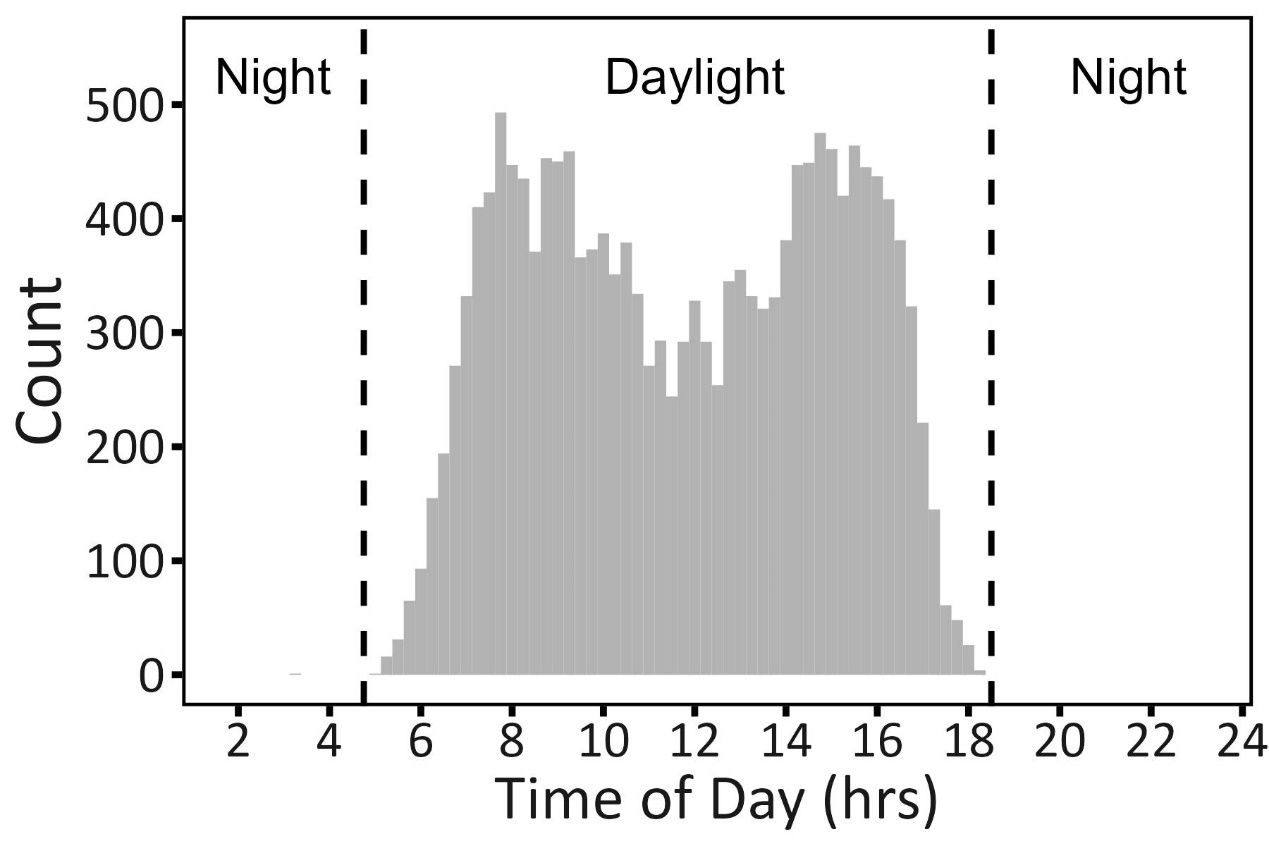


**Figure S1.** Frequency distribution of dives by hour of the day from dive data (1-second resolution) collected in 2015 and 2016 on Nazca boobies (see Methods in Chapter 5 of Howard 2021). Vertical dashed lines separate day and night periods (nautical dawn and dusk) at the Punta Cevallos colony.

## 2. Calculating Mass at Departure from Mass at Deployment

Wing Loading, a measure of structural body size used as an independent variable in models predicting foraging performance, was calculated from body mass at the start of a foraging absence (MassPre) divided by wing area (described above). To obtain MassPre, mass measurements from tagged birds were collected when loggers were deployed and then later corrected for the expected mass loss between deployment and departure (events that may be separated by several days). The body mass correction for MassPre used an empirical pattern of mass change during incubation from birds (n = 56, collected in 2014–2017) for which two mass measurements were collected during a single incubation bout (while fasting; Cherel et al. 1988; Prince et al. 1981). Mass loss of fasting birds is initially rapid (over the first few days), followed by a longer period of slower loss (Cherel et al. 1988). Based on this expected pattern, we fit threshold models to capture a two-phase pattern of mass change during incubation (as a function of “Incubation Day"). Threshold values were set at 1.0, 1.5, 2.0, 2.5, 3.0, 3.5, and 4.0 days, respectively, in candidate models whose relative performance was evaluated using AICc (Burnham and Anderson 2010). The performance of the threshold models was compared with that of an intercept-only model and of a model including only a linear change in mass with Incubation Day. We combined data from males and females, fitting an additional predictor of “Sex” to represent mean differences in size.

As expected, body mass decreased rapidly immediately after returning from a foraging trip (β = -0.186 [95% CI: -0.206, -0.165]; Figure S2), and more slowly after 1.5 days at the nest (β = -0.040 [95% CI: -0.024, 0.055]; Figure S2). The best-supported model describing variation in specific daily mass loss included a linear threshold function of time with a threshold at 1.5 days (Table S2). Conditional R^2^ (R^2^_c_) includes, and marginal R^2^ (R^2^_m_) excludes, the variance component for bird identity; R^2^_c_ for this top model is 0.93 and R^2^_m_ is 0.76, giving us confidence in using this relationship to estimate mass at departure. Additionally, some birds returned to the colony after completing a foraging absence after the last nightly check (20:00h), and their mass at arrival was not measured until the following morning (at logger retrieval). For these birds, we used this same relationship to add expected mass lost between arrival (from logger data) and logger retrieval to their mass at return.

**Table S2.** Changes in body mass over an incubation period in Nazca boobies. Model rankings compare alternative functions relating Incubation Day (days 1–7) to recorded body mass, including no effect, linear, and single-threshold (“One T”) functions. Models shown in bold are highly supported: they are within ΔAICc of 2 of the top model and are not a more complex version of a simpler, nested model with a lower AICc value. The number of parameters (*k*), AICc difference from the top model (ΔAICc), and Akaike weights (*ω_i_*) are reported. N = 112.

| **Time Model** | ***k*** | **ΔAICc** | **ωi** |
| --- | --- | --- | --- |
| **One T (1.5)** | **6** | **0.00** | **0.82** |
| One T (1) | 6 | 3.20 | 0.17 |
| One T (2) | 6 | 8.70 | 0.01 |
| One T (2.5) | 6 | 17.20 | <0.01 |
| One T (3) | 6 | 22.10 | <0.01 |
| One T (3.5) | 6 | 26.10 | <0.01 |
| One T (4) | 6 | 32.50 | <0.01 |
| Time | 5 | 54.40 | <0.01 |
| No Time | 4 | 157.70 | <0.01 |


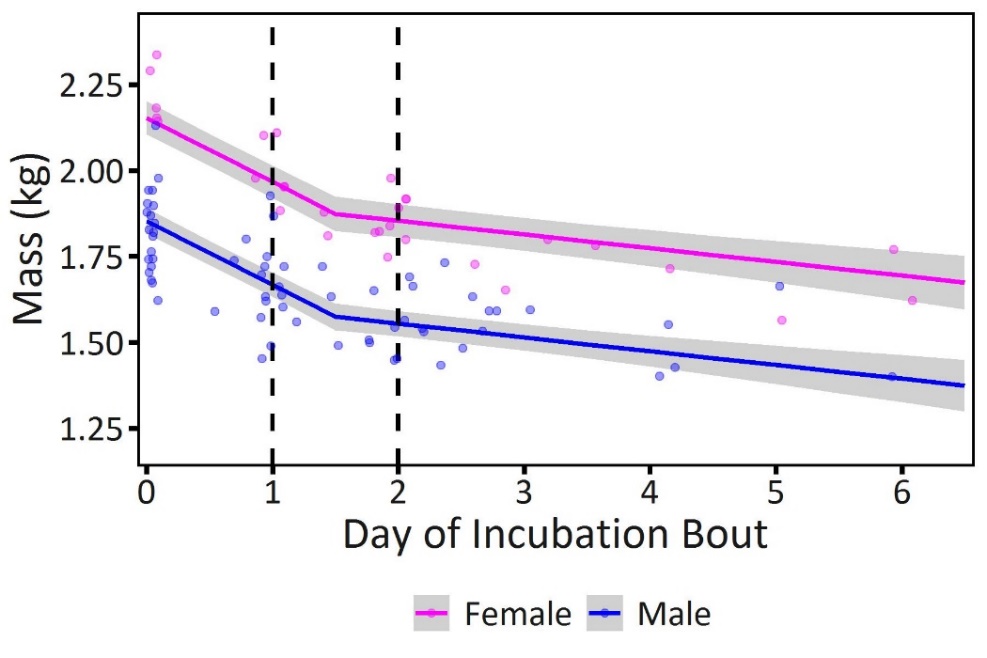


Figure S2. Two-phase decline in Nazca booby body mass during incubation (while fasting). Day “0” is the calendar day during which the bird returns to the nest after its foraging absence. Dashed lines indicate when birds were tagged with GPS loggers (on their second or third day of incubation).

## 3. EMbC Delimiters

Initially, the EMbC model output for each sex yielded speed delimiters much slower than expected for boobies to remain in the air (males: 1.31 m/s; females: 1.29 m/s). Speeds slower than these low thresholds identified resting behavior — Nazca boobies regularly spend nights on the water (Zavalaga et al. 2012) — with the majority of these slow speeds occurring at night (Figure S3), and were classified as resting. EMbC was then used secondarily to classify activity for the remaining logger locations; delimiters from the second model for each sex corresponded well with those from other booby species (Mendez et al. 2016, Lerma et al. 2020; Table S3).


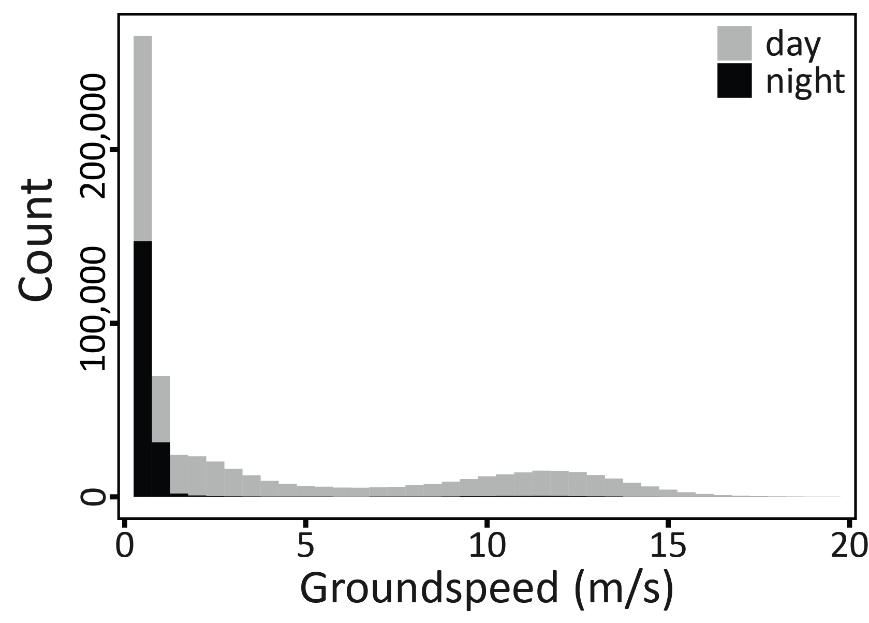


**Figure S3.** Frequency distribution of unfiltered, raw Groundspeed across 5-min. sampling intervals during foraging absences of male and female Nazca boobies. Color indicates the day versus night for each Groundspeed value**.**

**Table S3.** Delimiters from EMbC analysis classifying behaviors by speed (m/s) and turning angle (radians).

| **Sex** | **Behavior** | **Speed (m/s)** | | **Turning Angle (rad)** | |
| --- | --- | --- | --- | --- | --- |
|  |  | **Min** | **Max** | **Min** | **Max** |
| **Males** | Resting | 0.00 | 5.80 | 0.00 | 0.74 |
|  | Searching | 0.00 | 4.33 | 0.74 | 3.14 |
|  | Commuting | 5.80 | 37.50 | 0.00 | 0.38 |
|  | Relocating | 4.33 | 37.50 | 0.38 | 3.14 |
| **Females** | Resting | 0.00 | 7.12 | 0.00 | 0.34 |
|  | Searching | 0.00 | 4.97 | 0.34 | 3.14 |
|  | Commuting | 7.12 | 39.60 | 0.00 | 0.37 |
|  | Relocating | 4.97 | 39.60 | 0.37 | 3.14 |

## 4. Threshold Functions Describing Age Effects on Foraging

In Stage 1, we used AICc-based model selection to support age effects on foraging performance and to identify the best-supported age function for each foraging trait in each sex. Following results for breeding traits (Tompkins and Anderson 2019), the sexes were evaluated separately to allow a different optimal age function to be identified for males versus females. We fit models with no age effects and with linear, quadratic, and one- and two-threshold age functions and ranked their performance using AICc.

Two-threshold models divided the lifespan into early life, middle age, and late life based on the position of two threshold ages (T_1_, T_2_) and allowed the rate of change in performance with age to change across thresholds. We used the following two-threshold parameterization of the linear predictor for each foraging trait for individual *i* at age *j* (shown here for Mass Gain):

Mass Gain*_ij_* = α+β_1_Ag*e_ij_*+β_2_(Age*_ij_*–T_1_)_+_+β_3_(Age*_i_* –T_2_)_+_+β_4_x_4_…,+β_k_x_k_+*u_i_*+*u_season_*

Here threshold ages T_1_ and T_2_ were set for each candidate model and (Age*_ij_* – T_1_)_+_ represented the product of (Age*_ij_* – T_1_) and a logical function equal to 1 when Age*_ij_* > T_t_, and 0 otherwise. Candidate threshold ages were chosen following results of Tompkins and Anderson (2019, 2021); for T_1_, candidate ages were 6–10 years old for males and 6–8 years old for females and for T_2_ they were 13–17 years old for both sexes. Coefficient β_1_ estimates the slope of age on Mass Gain for young ages (ages ≤ T_1_), β_2_ estimates the change in slope after the first threshold age, and β_3_ estimates the change in slope after the second threshold age. The logical function switches the β_2_ term *off* for ages ≤ T_1_ and *on* for ages > than T_1_. Likewise, the logical function switches the β_3_ term *off* for ages ≤ T_2_ and *on* for ages > T_2_. In this example, the slope of age on Mass Gain before T_1_ is β_1_, the slope of age on Mass Gain between T_1_ and T_2_ is β_1_+β_2_, and the slope of age on Mass Gain after T_2_ is β_1_+β_2_+β_3_. Additional predictors (β_4_x_4_…, + β_k_x_k_) were included as described in the main text. Crossed random intercepts for individual identity (*u_i_*) and breeding season (*u_season_*) were included in all models.

Single threshold models tested for early-life improvement in the absence of late-life decline or vice versa and considered the same candidate threshold values as two-threshold models with the addition of ages 12 and 13.

## 5. Sea Surface Temperature

Gridded oceanographic data were accessed from the NOAA ERDDAP servers using the *rerddap* R package (Chamberlain 2016). We obtained 8-day composite values of SST, centered on individual dates of foraging during the five breeding seasons, for the Nazca booby foraging area from the product: Aqua MODIS satellite, SST, 0.025 degrees, Pacific Ocean Lon±180. The Nazca booby foraging area was defined as a polygon extending to the 95% quantile of longitudes and latitudes reached by Nazca boobies during this study (coordinates: 89.60°W to 85.73°W; 2.92°S to 0.54°N), which coincided generally with the foraging areas of Nazca boobies during chick rearing (Zavalaga et al. 2012). Temporal patterns in daily mean SST across this area (hereafter “SST”) were very similar to those from the larger polygon encompassing the furthest latitude and longitude coordinates reached (coordinates: 91.00°W to 82.65°W; 6.20°S to 2.00°N; r = 0.98, df = 360, p<0.0001). SST for some grid cells in the foraging area of this Nazca booby population was missing for some dates from the Aqua MODIS satellite product. For these dates, values were set to a mean value of SST (averaged across the foraging area as described above) from the 8-day window centered on the relevant date.

SST varies seasonally in and around the Galápagos archipelago (Palacios 2004). We first evaluated, and then removed, seasonal changes in SST during the three-month study period before using SST as a predictor of foraging traits. This allowed us to decouple environmental effects on Nazca booby foraging traits (effects of unusually cold, or unusually warm waters for a given time of year) from changes in foraging due to the advancement of breeding activities (e.g., due to being early versus late in the incubation period; e.g., Hedd et al. 2001, Howard et al. 2021). Seasonal changes in SST were evaluated with a linear mixed-effect model fitting extended Julian date (“Date”; expressed as daily increments across each breeding season’s two-year span) as a fixed effect, and a random effect of Breeding Season. Our model of SST supported seasonal increases in ocean temperature across the incubation period, so a residual SST was calculated by subtracting predicted SST (estimated from the fixed effect component of the model as predicted SST = -14.057+0.028⁎Date) from the raw SST (note that variable “SST” in subsequent results tables refers to daily SST averaged across this population’s foraging area after removing these seasonal changes).

## 6. Patterns of Covariation among Predictor Variables

We estimated pairwise correlations between predictor variables (Table S4) using a nonparametric correlation statistic (Kendall’s tau) because some predictor variables did not follow a normal distribution. When one variable was the factor Logger, we used a Wilcoxon rank sum test to test for differences in the distribution of each other predictor variable by Logger.

Table S4. Pairwise comparisons of predictors included together in Stage 2 analyses. Statistically significant results are in bold. Med. Date = Median Breeding Date.

| **Sex** | **Predictor 1** | **Predictor 2** | **Relationship** |
| --- | --- | --- | --- |
| **Males**  **N = 514** | Age | Wing Loading | Kendall τ_b_ = 0.04, p = 0.21 |
|  | Age | Date | Kendall τ_b_ = 0.04, p = 0.15 |
|  | Age | SST | Kendall τ_b_ = 0.05, p = 0.13 |
|  | Age | Med. Date | Kendall τ_b_ = 0.03, p = 0.31 |
|  | Age | Cloud | Kendall τ_b_ = 0.02, p = 0.45 |
|  | Age | Logger | W = 23078, N_1_ = 386, N_2_ = 128, p = 0.26 |
|  | Date | SST | **Kendall τ_b_ = 0.35, p < 0.01** |
|  | Date | Med. Date | **Kendall τ_b_ = 0.34, p < 0.01** |
|  | Date | Cloud | **Kendall τ_b_ = -0.16, p < 0.01** |
|  | Date | Logger | W = 22227, N_1_ = 386, N_2_ = 128, p = 0.09 |
|  | Date | Wing Loading | **Kendall τ_b_ = -0.07, p = 0.02** |
|  | Logger | Wing Loading | W = 32407, N_1_ = 386, N_2_ = 128, **p < 0.01** |
|  | Med. Date | Wing Loading | **Kendall τ_b_ = -0.40, p < 0.01** |
|  | SST | Wing Loading | **Kendall τ_b_ = -0.22, p < 0.01** |
|  | SST | Med. Date | **Kendall τ_b_ = 0.34, p < 0.01** |
|  | SST | Logger | W = 10005, N_1_ = 386, N_2_ = 128, **p < 0.01** |
|  | SST | Cloud | Kendall τ_b_ = -0.01, p = 0.71 |
|  | Logger | Cloud | W = 23192, N_1_ = 386, N_2_ = 128, p = 0.29 |
|  | Med. Date | Cloud | **Kendall τ_b_ = -0.07, p = 0.04** |
|  | Med. Date | Logger | W = 11372, N_1_ = 386, N_2_ = 128, **p < 0.01** |
| **Females**  **N = 446** | Age | Wing Loading | Kendall τ_b_ = -0.04, p = 0.22 |
|  | Age | Date | Kendall τ_b_ = 0.05, p = 0.12 |
|  | Age | SST | **Kendall τ_b_ = 0.07, p = 0.04** |
|  | Age | Med. Date | Kendall τ_b_ = 0.07, p = 0.06 |
|  | Age | Cloud | Kendall τ_b_ = -0.02, p = 0.62 |
|  | Age | Logger | W = 20874, N_1_ = 284, N_2_ = 162, p = 0.10 |
|  | Date | SST | **Kendall τ_b_ = 0.30, p < 0.01** |
|  | Date | Med. Date | **Kendall τ_b_ = 0.33, p < 0.01** |
|  | Date | Cloud | **Kendall τ_b_ = -0.14, p < 0.01** |
|  | Date | Logger | W = 15882, N_1_ = 284, N_2_ = 162, **p < 0.01** |
|  | Date | Wing Loading | **Kendall τ_b_ = -0.15, p < 0.01** |
|  | Logger | Wing Loading | W = 31035, N_1_ = 284, N_2_ = 162, **p < 0.01** |
|  | Med. Date | Wing Loading | **Kendall τ_b_ = -0.45, p < 0.01** |
|  | SST | Wing Loading | **Kendall τ_b_ = -0.23, p < 0.01** |
|  | SST | Med. Date | **Kendall τ_b_ = 0.34, p < 0.01** |
|  | SST | Logger | W = 7039, N_1_ = 284, N_2_ = 162, **p < 0.01** |
|  | SST | Cloud | Kendall τ_b_ = 0.20, p = 0.53 |
|  | Logger | Cloud | W = 23213, N_1_ = 284, N_2_ = 162, p =0.87 |
|  | Med. Date | Cloud | **Kendall τ_b_ = -0.07, p = 0.05** |
|  | Med. Date | Logger | W = 8326, N_1_ = 284, N_2_ = 162, **p < 0.01** |

## 7. Effects of Season on SST

In a *post hoc* analysis, we evaluated Breeding Season as a predictor of sea surface temperature (SST). SST, used here as a response variable, was the same variable used as a predictor of foraging traits in the main text (seasonal increases in SST have been removed, see p. 8). We included Breeding Season as a predictor to identify how much inter-annual variation (versus environmental changes within a Breeding Season) explains variation in these environmental variables. Consistent with the extreme El Niño in 2015 (Santoso et al. 2017), SST was much higher in 2015 compared to the other Breeding Seasons (Table S5, Figure S4). The five-level factor Breeding Season explained 80% of the variation in SST (r^2^ = 0.80).

Table S5***.*** Coefficient estimates, and SE, describing the effect of Breeding Season on SST (N = 401). Coefficients in bold have 95% CI that exclude zero.

|  | SST |
| --- | --- |
| **Coefficient** | **β (SE)** |
| Intercept | **-1.03 (0.07)** |
| Breeding Season (2012) | **-0.22 (0.10)** |
| Breeding Season (2014) | **1.19 (0.10)** |
| Breeding Season (2015) | **3.25 (0.10)** |
| Breeding Season (2016) | **0.82 (0.10)** |


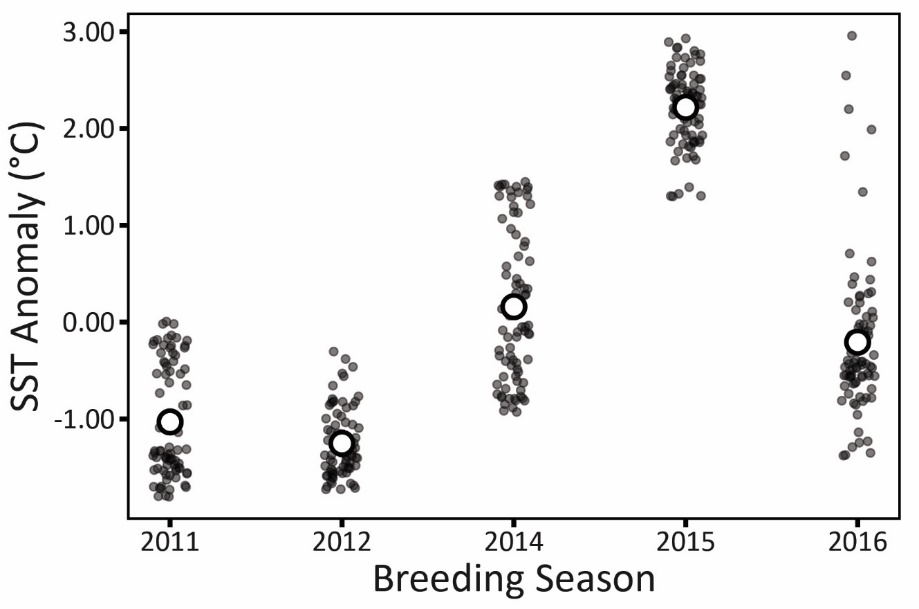


**Figure S4.** Variation in SST (after seasonal warming is removed; see Section 5) explained by Breeding Season. Large points show the model-predicted means ± 95% CI from the top model. Small points show the raw data; darker shades indicate overlap (jittered horizontally).

## 8. Male Stage 1 Complete Model Results

**Table S6.** Stage 1 age effects on male foraging traits: log(Mass Gain/hr), Mass Gain, sqrt(Absence Duration), sqrt(Total Distance), and sqrt(Time Searching). Model rankings compare alternative parameterizations of age effects, including no age, linear age, quadratic age, and single-threshold (“One T”) or two-threshold functions (“Two T”). Models shown in bold are considered to be highly supported: they are within ΔAICc of 2 of the top model and are not a more complex version of a simpler, nested model with a lower AICc value. The number of parameters (*k*), AICc difference from the top model (ΔAICc), and Akaike weights (*ω_i_*) are reported. ID and DepartGroup random effects and additional fixed-effect predictors were held constant across all models (main text Methods).

|  |  | **log(Mass Gain/hr)** | |  | **Mass Gain** | |  | **sqrt(Absence Duration)** | |  | **sqrt(Total Distance)** | | **sqrt(Time Searching)** | | |  |
| --- | --- | --- | --- | --- | --- | --- | --- | --- | --- | --- | --- | --- | --- | --- | --- | --- |
| **Sample Size** |  | 480 | |  | 492 | |  | 514 | |  | 505 | | 505 | |  |  |
| **Age Model** | ***k*** | **ΔAICc** | **ωi** |  | **ΔAICc** | **ωi** |  | **ΔAICc** | **ωi** |  | **ΔAICc** | **ωi** | **ΔAICc** | **ωi** | | |
| No Age | 11 | **1.88** | **0.04** |  | 3.60 | 0.02 |  | **0.00** | **0.12** |  | **0.00** | **0.26** | **0.00** | **0.11** | | |
| Age | 12 | **0.00** | **0.11** |  | **0.00** | **0.13** |  | 0.78 | 0.08 |  | 2.06 | 0.09 | 0.20 | 0.10 | | |
| Age + Age^2^ | 13 | 1.37 | 0.06 |  | 2.00 | 0.05 |  | 1.84 | 0.05 |  | 3.89 | 0.04 | 1.34 | 0.06 | | |
| One T (7) | 13 | 1.79 | 0.04 |  | 1.93 | 0.05 |  | 2.45 | 0.03 |  | 3.73 | 0.04 | 2.31 | 0.03 | | |
| One T (8) | 13 | 1.75 | 0.05 |  | 2.01 | 0.05 |  | 2.88 | 0.03 |  | 4.17 | 0.03 | 2.28 | 0.03 | | |
| One T (9) | 13 | 1.40 | 0.05 |  | 2.10 | 0.04 |  | 2.40 | 0.04 |  | 4.00 | 0.04 | 1.88 | 0.04 | | |
| One T (10) | 13 | 1.27 | 0.06 |  | 2.10 | 0.04 |  | 1.75 | 0.05 |  | 3.81 | 0.04 | 1.48 | 0.05 | | |
| One T (12) | 13 | 1.18 | 0.06 |  | 2.07 | 0.05 |  | 1.34 | 0.06 |  | 3.82 | 0.04 | 1.29 | 0.06 | | |
| One T (13) | 13 | 1.49 | 0.05 |  | 2.08 | 0.05 |  | 1.89 | 0.05 |  | 4.06 | 0.03 | 1.84 | 0.04 | | |
| One T (14) | 13 | 1.71 | 0.05 |  | 2.10 | 0.04 |  | 1.82 | 0.05 |  | 4.04 | 0.03 | 1.93 | 0.04 | | |
| One T (15) | 13 | 1.78 | 0.04 |  | 1.94 | 0.05 |  | 2.01 | 0.04 |  | 3.96 | 0.04 | 1.83 | 0.04 | | |
| One T (16) | 13 | 1.75 | 0.05 |  | 1.72 | 0.05 |  | 1.89 | 0.05 |  | 3.74 | 0.04 | 1.52 | 0.05 | | |
| One T (17) | 13 | 1.71 | 0.05 |  | 1.64 | 0.06 |  | 1.98 | 0.04 |  | 3.77 | 0.04 | 1.32 | 0.06 | | |
| Two T (7, 14) | 14 | 3.72 | 0.02 |  | 4.04 | 0.02 |  | 2.57 | 0.03 |  | 5.37 | 0.02 | 3.96 | 0.01 | | |
| Two T (7, 15) | 14 | 3.75 | 0.02 |  | 3.97 | 0.02 |  | 3.01 | 0.03 |  | 5.30 | 0.02 | 3.85 | 0.02 | | |
| Two T (7, 16) | 14 | 3.72 | 0.02 |  | 3.78 | 0.02 |  | 2.95 | 0.03 |  | 5.02 | 0.02 | 3.53 | 0.02 | | |
| Two T (7, 17) | 14 | 3.67 | 0.02 |  | 3.70 | 0.02 |  | 3.19 | 0.02 |  | 5.13 | 0.02 | 3.33 | 0.02 | | |
| Two T (8, 14) | 14 | 3.77 | 0.02 |  | 4.11 | 0.02 |  | 3.15 | 0.02 |  | 6.06 | 0.01 | 3.95 | 0.01 | | |
| Two T (8, 15) | 14 | 3.80 | 0.02 |  | 4.06 | 0.02 |  | 3.63 | 0.02 |  | 5.97 | 0.01 | 3.84 | 0.02 | | |
| Two T (8, 16) | 14 | 3.76 | 0.02 |  | 3.84 | 0.02 |  | 3.56 | 0.02 |  | 5.68 | 0.02 | 3.48 | 0.02 | | |
| Two T (8, 17) | 14 | 3.71 | 0.02 |  | 3.76 | 0.02 |  | 3.82 | 0.02 |  | 5.76 | 0.01 | 3.29 | 0.02 | | |
| Two T (9, 14) | 14 | 3.51 | 0.02 |  | 4.22 | 0.02 |  | 3.84 | 0.02 |  | 6.12 | 0.01 | 3.98 | 0.01 | | |
| Two T (9, 15) | 14 | 3.50 | 0.02 |  | 3.92 | 0.02 |  | 4.12 | 0.01 |  | 6.07 | 0.01 | 3.91 | 0.02 | | |
| Two T (9, 16) | 14 | 3.52 | 0.02 |  | 3.56 | 0.02 |  | 4.00 | 0.02 |  | 5.85 | 0.01 | 3.64 | 0.02 | | |
| Two T (9, 17) | 14 | 3.51 | 0.02 |  | 3.54 | 0.02 |  | 4.09 | 0.02 |  | 5.88 | 0.01 | 3.43 | 0.02 | | |
| Two T (10, 14) | 14 | 3.16 | 0.02 |  | 4.08 | 0.02 |  | 3.84 | 0.02 |  | 5.73 | 0.01 | 3.32 | 0.02 | | |
| Two T (10, 15) | 14 | 3.21 | 0.02 |  | 3.39 | 0.02 |  | 3.87 | 0.02 |  | 5.91 | 0.01 | 3.56 | 0.02 | | |
| Two T (10, 16) | 14 | 3.34 | 0.02 |  | 2.91 | 0.03 |  | 3.81 | 0.02 |  | 5.84 | 0.01 | 3.53 | 0.02 | | |
| Two T (10, 17) | 14 | 3.39 | 0.02 |  | 3.07 | 0.03 |  | 3.80 | 0.02 |  | 5.84 | 0.01 | 3.36 | 0.02 | | |

**
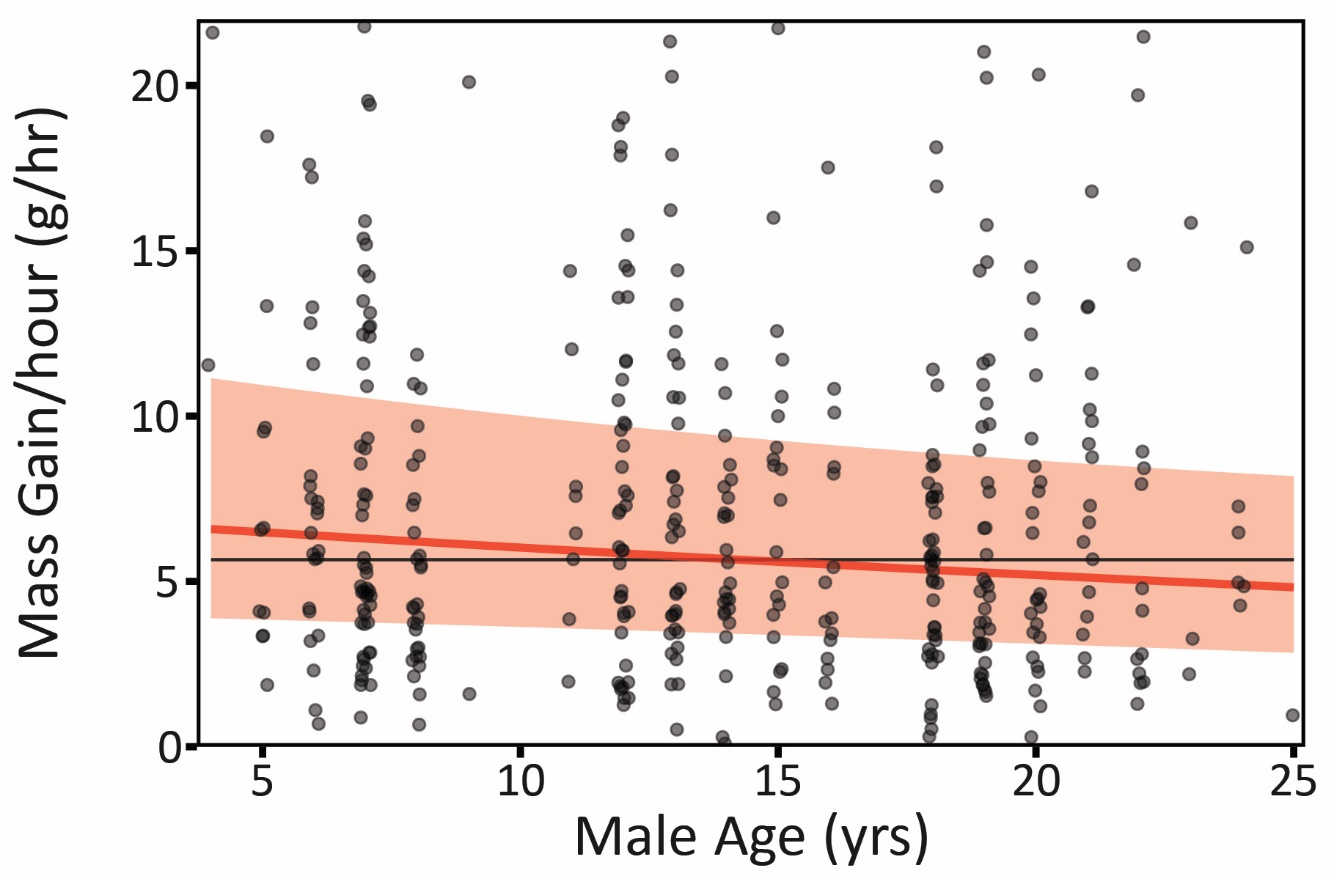
**

**Figure S5.** Age effects on foraging traits were supported weakly for male Mass Gain/hr. The red line is the model prediction from the top model from Stage 1 holding continuous predictors (other than age) at their mean value and factor Breeding Season at 2014 (a “typical” year) and factor Logger at i-gotU GT-120; the red shaded region is the 95% CI from the top model from Stage 1. Black lines are predictions from models falling within 2 ΔAICc of the top model (the top model set), illustrating model selection uncertainty in the form of the age function. Points show the raw data; darker shades indicate overlap (jittered horizontally). We modeled log(Mass Gain/hr), but back-transformed the predicted values to show effect size.

## 9. Female Stage 1 Complete Model Results

**Table S7.** Stage 1 age effects on female foraging traits: log(Mass Gain/hr), Mass Gain, sqrt(Absence Duration), sqrt(Total Distance), and sqrt(Time Searching). Results are presented as for Table S6.

|  |  | | **Log(Mass Gain/hr)** | | |  | | **Mass Gain** | | |  | | **sqrt(Absence Duration)** | | |  | | **sqrt(Total Distance)** | | | **sqrt(Time Searching)** | | | |  |  |
| --- | --- | --- | --- | --- | --- | --- | --- | --- | --- | --- | --- | --- | --- | --- | --- | --- | --- | --- | --- | --- | --- | --- | --- | --- | --- | --- |
| **Sample Size** | |  | | 450 | | |  | | 461 | | |  | | 446 | | |  | | 428 | | | 428 | | | | |
| **Age Model** | | ***k*** | | **ΔAICc** | **ωi** | |  | | **ΔAICc** | **ωi** | |  | | **ΔAICc** | **ωi** | |  | | **ΔAICc** | **ωi** | |  | **ΔAICc** | **ωi** | |  |
| No Age | | 11 | | **1.92** | **0.04** | |  | | **0.59** | **0.11** | |  | | 4.46 | 0.02 | |  | | 3.34 | 0.03 | |  | 3.41 | 0.02 | |  |
| Age | | 12 | | 2.11 | 0.04 | |  | | 2.25 | 0.05 | |  | | 4.57 | 0.02 | |  | | 4.88 | 0.01 | |  | **1.79** | **0.05** | |  |
| Age + Age^2^ | | 13 | | **0.75** | **0.07** | |  | | 2.15 | 0.05 | |  | | **0.00** | **0.15** | |  | | **0.00** | **0.14** | |  | **1.38** | **0.06** | |  |
| One T (6) | | 13 | | **1.41** | **0.05** | |  | | 3.35 | 0.03 | |  | | 6.07 | 0.01 | |  | | 5.28 | 0.01 | |  | 3.80 | 0.02 | |  |
| One T (7) | | 13 | | **0.43** | **0.08** | |  | | 3.01 | 0.03 | |  | | 5.21 | 0.01 | |  | | 3.83 | 0.02 | |  | 3.79 | 0.02 | |  |
| One T (8) | | 13 | | **0.79** | **0.07** | |  | | 2.11 | 0.05 | |  | | 3.71 | 0.02 | |  | | 2.56 | 0.04 | |  | 3.87 | 0.02 | |  |
| One T (12) | | 13 | | **0.00** | **0.10** | |  | | **0.00** | **0.14** | |  | | **0.11** | **0.15** | |  | | **0.46** | **0.11** | |  | 2.17 | 0.04 | |  |
| One T (13) | | 13 | | **0.91** | **0.07** | |  | | 0.62 | 0.10 | |  | | **0.81** | **0.10** | |  | | **0.70** | **0.10** | |  | 2.13 | 0.04 | |  |
| One T (14) | | 13 | | **1.85** | **0.04** | |  | | 1.36 | 0.07 | |  | | **1.72** | **0.07** | |  | | **1.79** | **0.06** | |  | 1.99 | 0.05 | |  |
| One T (15) | | 13 | | 2.41 | 0.03 | |  | | 1.72 | 0.06 | |  | | 2.37 | 0.05 | |  | | 2.47 | 0.04 | |  | 1.88 | 0.05 | |  |
| One T (16) | | 13 | | 2.67 | 0.03 | |  | | 2.52 | 0.04 | |  | | 2.40 | 0.05 | |  | | 2.57 | 0.04 | |  | **1.51** | **0.06** | |  |
| One T (17) | | 13 | | 2.50 | 0.03 | |  | | 3.59 | 0.02 | |  | | **1.10** | **0.09** | |  | | **1.39** | **0.07** | |  | **0.00** | **0.12** | |  |
| Two T (6, 14) | | 14 | | 2.37 | 0.03 | |  | | 3.21 | 0.03 | |  | | 3.84 | 0.02 | |  | | 3.56 | 0.02 | |  | 3.43 | 0.02 | |  |
| Two T (6, 15) | | 14 | | 2.65 | 0.03 | |  | | 3.46 | 0.03 | |  | | 4.45 | 0.02 | |  | | 4.04 | 0.02 | |  | 3.43 | 0.02 | |  |
| Two T (6, 16) | | 14 | | 2.74 | 0.03 | |  | | 4.10 | 0.02 | |  | | 4.44 | 0.02 | |  | | 4.01 | 0.02 | |  | 3.12 | 0.03 | |  |
| Two T (6, 17) | | 14 | | 2.51 | 0.03 | |  | | 4.96 | 0.01 | |  | | 3.12 | 0.03 | |  | | 2.79 | 0.04 | |  | 1.58 | 0.06 | |  |
| Two T (7, 14) | | 14 | | 2.07 | 0.04 | |  | | 3.32 | 0.03 | |  | | 3.82 | 0.02 | |  | | 3.35 | 0.03 | |  | 2.71 | 0.03 | |  |
| Two T (7, 15) | | 14 | | 2.22 | 0.03 | |  | | 3.55 | 0.02 | |  | | 4.38 | 0.02 | |  | | 3.73 | 0.02 | |  | 2.83 | 0.03 | |  |
| Two T (7, 16) | | 14 | | 2.25 | 0.03 | |  | | 4.13 | 0.02 | |  | | 4.33 | 0.02 | |  | | 3.63 | 0.02 | |  | 2.57 | 0.03 | |  |
| Two T (7, 17) | | 14 | | 2.05 | 0.04 | |  | | 4.84 | 0.01 | |  | | 3.02 | 0.03 | |  | | 2.46 | 0.04 | |  | 0.95 | 0.08 | |  |
| Two T (8, 14) | | 14 | | 2.80 | 0.03 | |  | | 3.30 | 0.03 | |  | | 3.80 | 0.02 | |  | | 3.46 | 0.03 | |  | 3.03 | 0.03 | |  |
| Two T (8, 15) | | 14 | | 2.88 | 0.02 | |  | | 3.48 | 0.02 | |  | | 4.26 | 0.02 | |  | | 3.74 | 0.02 | |  | 3.19 | 0.03 | |  |
| Two T (8, 16) | | 14 | | 2.88 | 0.02 | |  | | 3.88 | 0.02 | |  | | 4.16 | 0.02 | |  | | 3.61 | 0.02 | |  | 2.94 | 0.03 | |  |
| Two T (8, 17) | | 14 | | 2.75 | 0.03 | |  | | 4.22 | 0.02 | |  | | 2.92 | 0.04 | |  | | 2.57 | 0.04 | |  | 1.23 | 0.07 | |  |

## 10. Post hoc Analysis of Age by Sex Interaction

**Table S8**. Coefficient estimates from a model examining sex-specific differences in aging. Age was standardized (0 mean, 1 sd) so that the coefficient on Sex estimates the difference between males and females at the mean age within each dataset. The evaluated fixed-effect model was:

foraging trait ~Date+Logger+Wing Loading+Season+Age+Age^2^+Sex+Age*Sex+Age^2^*Sex. Random effects included ID and DepartGroup. Coefficients in bold have 95% CIs that exclude zero. Conditional R^2^ (R^2^_c_) includes, and marginal R^2^ (R^2^_m_) excludes, the variance explained by the random effects.

|  | **Log(Mass Gain/hr)** |  | **Mass Gain** |  | **sqrt(Absence Duration)** |  | **sqrt(Total Distance)** |  | **sqrt(Time Searching)** |
| --- | --- | --- | --- | --- | --- | --- | --- | --- | --- |
| Sample Size | 930 |  | 953 |  | 960 |  | 933 |  | 933 |
| **Coefficient** | **β [95% CI]** |  | **β [95% CI]** |  | **β [95% CI]** |  | **β [95% CI]** |  | **β [95% CI]** |
| Intercept | **0.97 [0.83, 1.11]** |  | **456.59 [423.28, 491.12]** |  | **5.81 [5.14, 6.45]** |  | **25.35 [21.59, 29.04]** |  | **1.98 [1.77, 2.18]** |
| Date | **0.08 [0.02, 0.14]** |  | **27.20 [12.55, 42.54]** |  | -0.15 [-0.43, 0.13] |  | -0.47 [-1.98, 0.96] |  | -0.08 [-0.17, 0.01] |
| Logger (600) | **-0.08 [-0.15, -0.01]** |  | 1.74 [-25.24, 27.08] |  | **0.52 [0.16, 0.87]** |  | **1.70 [0.40, 3.01]** |  | **0.19 [0.09, 0.29]** |
| Wing Loading | 0.01 [-0.02, 0.04] |  | **-76.9 [-88.21, -64.67]** |  | **-0.64 [-0.8, -0.49]** |  | **-2.09 [-2.67, -1.54]** |  | **-0.21 [-0.26, -0.17]** |
| Season (2012) | -0.07 [-0.24, 0.11] |  | -25.05 [-64.97, 15.06] |  | -0.1 [-0.91, 0.74] |  | -0.74 [-5.54, 4.09] |  | -0.13 [-0.39, 0.13] |
| Season (2014) | -0.09 [-0.3, 0.11] |  | **-128.29 [-176.41, -79.3]** |  | -0.66 [-1.62, 0.34] |  | -2.87 [-8.38, 2.73] |  | -0.12 [-0.42, 0.20] |
| Season (2015) | 0.29 [0.08, 0.49] |  | **-137.21 [-186.04, -88.8]** |  | **-1.94 [-2.89, -0.97]** |  | **-9.8 [-15.25, -4.25]** |  | **-0.35 [-0.64, -0.04]** |
| Season (2016) | -0.16 [-0.35, 0.02] |  | **-115.20 [-161.01, -69.05]** |  | 0.25 [-0.61, 1.14] |  | 1.7 [-3.17, 6.62] |  | 0.09 [-0.18, 0.37] |
| Age | -0.03 [-0.06, 0.00] |  | -6.73 [-19.40, 5.98] |  | 0.16 [-0.02, 0.33] |  | 0.43 [-0.21, 1.07] |  | **0.07 [0.02, 0.13]** |
| Age^2^ | **-0.05 [-0.08, -0.01]** |  | -12.96 [-28.53, 2.45] |  | **0.28 [0.07, 0.48]** |  | **1.09 [0.32, 1.84]** |  | **0.08 [0.02, 0.14]** |
| Sex (♂) | **-0.09 [-0.16, -0.02]**  **(0.623)** |  | **-80.70 [-109.83, -52.81]** |  | 0.29 [-0.09, 0.67] |  | -0.58 [-1.97, 0.79] |  | **-0.40 [-0.51, -0.29]** |
| Age*Sex (♂) | -0.01 [-0.05, 0.04]  0.388] |  | -5.79 [-24.04, 12.24] |  | -0.03 [-0.28, 0.21] |  | -0.29 [-1.18, 0.59] |  | -0.04 [-0.11, 0.03] |
| Age^2^*Sex (♂) | 0.03 [-0.02, 0.09] |  | 16.78 [-4.75, 38.91] |  | -0.17 [-0.45, 0.12] |  | -0.86 [-1.91, 0.20] |  | -0.05 [-0.14, 0.03] |
|  |  |  |  |  |  |  |  |  |  |
|  | **Variance** |  | **Variance** |  | **Variance** |  | **Variance** |  | **Variance** |
| ID | 0.01 |  | 812.15 |  | 0.23 |  | 1.14 |  | 0.00 |
| DepartGroup | 0.02 |  | 830.38 |  | 0.51 |  | 20.29 |  | 0.05 |
| Residual | 0.12 |  | 19,022.35 |  | 3.24 |  | 44.25 |  | 0.28 |
|  |  |  |  |  |  |  |  |  |  |
| **R^2^_m_** | 0.19 |  | 0.23 |  | 0.21 |  | 0.25 |  | 0.30 |
| **R^2^_c_** | 0.36 |  | 0.30 |  | 0.36 |  | 0.50 |  | 0.42 |

## 11. Male and Female Stage 2 Coefficient Estimates

**Table S9**. Coefficient estimates from the top model describing variation in male and female foraging traits (Stage 2 analyses). Coefficients in bold have 95% CIs that exclude zero. “(Age - T_1_)_+_” denotes the product of (Age - T_1_) times an indicator function equaling 1 when Age>T_1_  and 0 otherwise. Threshold age (T_1_) is 12 for female Mass Gain/hr and 17 for female Time Searching. Conditional R^2^ (R^2^_c_) includes, and marginal R^2^ (R^2^_m_) excludes, the variance explained by the random effects. Med. Date = Median Breeding Date.

|  |  | **log(Mass Gain/hr)** | |  | **Mass Gain** | | |  | **sqrt(Absence Duration)** | | |  | **sqrt(Total Distance)** | | |  | **Sqrt(Time Searching)** | | | |  |
| --- | --- | --- | --- | --- | --- | --- | --- | --- | --- | --- | --- | --- | --- | --- | --- | --- | --- | --- | --- | --- | --- |
|  | Sample Size | 467 | |  | 479 | | |  | 514 | | |  | 505 | | |  | 505 | | | |  |
|  | **Coefficient** | **β (SE)** | |  | **β (SE)** | | |  | **β (SE)** | | |  | **β (SE)** | | |  | **β (SE)** | | | |  |
| **Males** | Intercept | **0.97 (0.06)** | |  | **347.22 (19.35)** | | |  | **5.59 (0.19)** | | |  | **22.14 (0.97)** | | |  | **1.47 (0.05)** | | | |  |
|  | Date | 0.05 (0.04) | |  | **21.17 (10.83)** | | |  | 0.01 (0.18) | | |  | -0.05 (0.87) | | |  | -0.03 (0.05) | | | |  |
|  | Logger (600) | -0.13 (0.05) | |  | -17.5 (16.84) | | |  | **0.65 (0.26)** | | |  | **2.16 (0.96)** | | |  | **0.19 (0.06)** | | | |  |
|  | Wing Loading | - | |  | **-71.51 (7.37)** | | |  | **-0.66 (0.11)** | | |  | **-2.20 (0.39)** | | |  | **-0.18 (0.03)** | | | |  |
|  | SST | **0.11 (0.03)** | |  | - | | |  | **-0.54 (0.14)** | | |  | **-2.64 (0.68)** | | |  | **-0.09 (0.04)** | | | |  |
|  | Med. Date | - | |  | **-31.77 (11.6)** | | |  | - | | |  | - | | |  | - | | | |  |
|  | Age | -0.01 (0.00) | |  | **-3.14 (1.14)** | | |  | - | | |  | - | | |  | - | | | |  |
|  |  |  | |  |  | | |  |  | | |  |  | | |  |  | | | |  |
|  |  | **Variance** | |  | **Variance** | | |  | **Variance** | | |  | **Variance** | | |  | **Variance** | | | |  |
|  | ID | 0.00 | |  | 0.00 | | |  | 0.28 | | |  | 2.96 | | |  | 0.03 | | | |  |
|  | DepartGroup | 0.04 | |  | 2,051.45 | | |  | 0.57 | | |  | 19.58 | | |  | 0.04 | | | |  |
|  | Residual | 0.14 | |  | 16,179.65 | | |  | 3.64 | | |  | 45.95 | | |  | 0.18 | | | |  |
|  |  |  | |  |  | | |  |  | | |  |  | | |  |  | | |  |  |
|  | **R^2^_m_** | 0.13 | |  | 0.20 | | |  | 0.14 | | |  | 0.17 | | |  | 0.15 | | | |  |
|  | **R^2^_c_** | 0.34 | |  | 0.29 | | |  | 0.31 | | |  | 0.44 | | |  | 0.39 | | | |  |
|  |  | **Mass Gain/hr** | |  | **Mass Gain** | | |  | **Absence Duration** | | |  | **Total Distance** | | |  | **Time Searching** | | | |  |
|  | Sample Size | 428 | |  | 454 | | |  | 424 | | |  | 406 | | |  | 406 | | | |  |
|  | **Coefficient** | **β (SE)** | |  | **β (SE)** | | |  | **β (SE)** | | |  | **β (SE)** | | |  | **β (SE)** | | | |  |
| **Females** | Intercept | **0.85 (0.08)** | |  | **338.25 (35.25)** | | |  | **6.56 (0.61)** | | |  | **27.98 (2.46)** | | |  | **1.94 (0.12)** | | | |  |
|  | Date | 0.05 (0.03) | |  | 14.77 (11.22) | | |  | -0.09 (0.17) | | |  | -0.12 (0.91) | | |  | -0.08 (0.05) | | | |  |
|  | Logger (600) | -0.09 (0.04) | |  | -24.12 (19.73) | | |  | **0.52 (0.24)** | | |  | **1.95 (0.93)** | | |  | **0.26 (0.08)** | | | |  |
|  | Wing Loading | **0.07 (0.02)** | |  | **-67.73 (8.53)** | | |  | **-0.77 (0.10)** | | |  | **-2.56 (0.40)** | | |  | **-0.32 (0.04)** | | | |  |
|  | SST | -0.01 (0.06) | |  | **-27.05 (9.22)** | | |  | **-0.54 (0.14)** | | |  | **-2.87 (0.72)** | | |  | **-0.10 (0.04)** | | | |  |
|  | Age | 0.01 (0.01) | |  | 5.34 (3.63) | | |  | **-0.20 (0.10)** | | |  | **-0.78 (0.38)** | | |  | -0.01 (0.01) | | | |  |
|  | Age^2^ | - | |  | - | | |  | **0.01 (0.00)** | | |  | 0.03 (0.01) | | |  | - | | | |  |
|  | (Age - T_1_)_+_ | -0.03 (0.01) | |  | -11.64 (5.64) | | |  | - | | |  | - | | |  | 0.06 (0.04) | | | |  |
|  | Age⁎SST | | 0.01 (0.01) | | |  | - | | |  | - | | |  | - | | |  | - | | |
|  | (Age - T_1_)_+_⁎SST | | 0.00 (0.01)* | | |  | - | | |  | - | | |  | - | | |  | - | | |
|  |  |  | |  |  | | |  |  | | |  |  | | |  |  | | | |  |
|  |  | **Variance** | |  | **Variance** | | |  | **Variance** | | |  | **Variance** | | |  | **Variance** | | | |  |
|  | ID | 0.00 | |  | 684.97 | | |  | 0.01 | | |  | 2.96 | | |  | 0.00 | | | |  |
|  | DepartGroup | 0.02 | |  | 1,431.28 | | |  | 0.52 | | |  | 19.58 | | |  | 0.04 | | | |  |
|  | Residual | 0.10 | |  | 22,705.24 | | |  | 2.88 | | |  | 45.95 | | |  | 0.38 | | | |  |
|  |  |  | |  |  | | |  |  | | |  |  | | |  |  | | | |  |
|  | **R^2^_m_** | 0.16 | |  | 0.18 | | |  | 0.21 | | |  | 0.21 | | |  | 0.24 | | | |  |
|  | **R^2^_c_** | 0.32 | |  | 0.25 | | |  | 0.34 | | |  | 0.49 | | |  | 0.32 | | | |  |

*Coefficient describes the change in the strength of the interaction after the threshold.  The total interaction strength after age 12 is calculated as the sum of the coefficient on Age*SST and that on (Age - T_1_)_+_⁎SST and is reported in the main text.

## 12. Male Complete Stage 2 AICc Model Rankings

**Table S10**. Models from Stage 2 analyses explaining variation in response variables, ranked by AICc. Models shown in bold are considered to be highly supported: they are within ΔAICc of 2 of the top model and are not a more complex version of a simpler, nested model. Random effects of DepartGroup and ID appeared in all models. The number of parameters (*k*), AICc difference from the top model (ΔAICc), and Akaike weights (*ω_i_*) are reported. Med. Date = Median Breeding Date.

| **log(Mass Gain/hr)** | ***k*** | **ΔAICc** | **ωi** |
| --- | --- | --- | --- |
| **Date + Logger + Age + SST** | **8** | **0.00** | **0.51** |
| Date + Logger + Age + Wing Loading + SST | 9 | 1.94 | 0.19 |
| Date + Logger + Age + SST + Age*SST | 9 | 2.02 | 0.18 |
| Date + Logger + Age + Wing Loading + SST + Age*SST | 10 | 3.97 | 0.07 |
| Date + Logger + Age + Cloud Cover | 8 | 7.23 | 0.01 |
| Date + Logger + Age + Wing Loading + Cloud Cover | 9 | 7.86 | 0.01 |
| Date + Logger + Age + Cloud Cover + Age*Cloud Cover | 9 | 8.99 | 0.01 |
| Date + Logger + Age + Wing Loading + Cloud Cover + Age*Cloud Cover | 10 | 9.49 | 0.00 |
| Date + Logger + Age | 7 | 9.61 | 0.00 |
| Date + Logger + Age + Wing Loading | 8 | 10.54 | 0.00 |
| Date + Logger + Age + Med. Date | 8 | 11.61 | 0.00 |
| Date + Logger + Age + Wing Loading + Med. Date | 9 | 12.29 | 0.00 |
| Date + Logger + Age + Med. Date + Age*Med. Date | 9 | 13.44 | 0.00 |
| Date + Logger + Age + Wing Loading + Med. Date + Age*Med. Date | 10 | 14.01 | 0.00 |
| **Mass Gain** | ***k*** | **ΔAICc** | **ωi** |
| **Date + Logger + Age + Wing Loading + Med. Date** | **9** | **0.00** | **0.52** |
| Date + Logger + Age + Wing Loading + Med. Date + Age*Med. Date | 10 | 1.03 | 0.31 |
| Date + Logger + Age + Wing Loading + SST | 9 | 4.32 | 0.06 |
| Date + Logger + Age + Wing Loading + SST + Age*SST | 10 | 4.69 | 0.05 |
| Date + Logger + Age + Wing Loading | 8 | 5.61 | 0.03 |
| Date + Logger + Age + Wing Loading + Cloud Cover | 9 | 6.98 | 0.02 |
| Date + Logger + Age + Wing Loading + Cloud Cover + Age*Cloud Cover | 10 | 8.69 | 0.01 |
| Date + Logger + Age | 7 | 82.09 | 0.00 |
| Date + Logger + Age + SST | 8 | 83.85 | 0.00 |
| Date + Logger + Age + Med. Date | 8 | 83.88 | 0.00 |
| Date + Logger + Age + Cloud Cover | 8 | 84.06 | 0.00 |
| Date + Logger + Age + SST + Age*SST | 9 | 84.07 | 0.00 |
| Date + Logger + Age + Cloud Cover + Age*Cloud Cover | 9 | 84.59 | 0.00 |
| Date + Logger + Age + Med. Date + Age*Med. Date | 9 | 85.85 | 0.00 |
| **sqrt(Absence Duration)** | ***k*** | **ΔAICc** | **ωi** |
| **Date + Logger + Wing Loading + SST** | **8** | **0.00** | **0.99** |
| Date + Logger + Wing Loading | 7 | 11.11 | 0.00 |
| Date + Logger + Wing Loading + Cloud Cover | 8 | 12.47 | 0.00 |
| Date + Logger + Wing Loading + Med. Date | 8 | 12.94 | 0.00 |
| Date + Logger + SST | 7 | 33.62 | 0.00 |
| Date | 6 | 37.88 | 0.00 |
| Date + Logger + Cloud Cover | 7 | 38.24 | 0.00 |
| Date + Logger + Med. Date | 7 | 38.99 | 0.00 |
| **sqrt(Total Distance)** | ***k*** | **ΔAICc** | **ωi** |
| **Date + Logger + Wing Loading + SST** | **8** | **0.00** | **0.98** |
| Date + Logger + Wing Loading + Cloud Cover | 8 | 8.09 | 0.02 |
| Date + Logger + Wing Loading | 7 | 11.65 | 0.00 |
| Date + Logger + Wing Loading + Med. Date | 8 | 13.32 | 0.00 |
| Date + Logger + SST | 7 | 28.69 | 0.00 |
| Date + Logger + Cloud Cover | 7 | 29.85 | 0.00 |
| Date | 6 | 35.22 | 0.00 |
| Date + Logger + Med. Date | 7 | 37.04 | 0.00 |
| **sqrt(Time Searching)** | ***k*** | **ΔAICc** | **ωi** |
| **Date + Logger + Wing Loading + SST** | **8** | **0.00** | **0.83** |
| Date + Logger + Wing Loading | 7 | 4.66 | 0.08 |
| Date + Logger + Wing Loading + Cloud Cover | 8 | 5.13 | 0.06 |
| Date + Logger + Wing Loading + Med. Date | 8 | 6.65 | 0.03 |
| Date + Logger + Cloud Cover | 7 | 45.54 | 0.00 |
| Date + Logger + SST | 7 | 46.56 | 0.00 |
| Date + Logger + Med. Date | 7 | 46.69 | 0.00 |
| Date | 6 | 46.73 | 0.00 |

## 13. Female Complete Stage 2 AICc Model Rankings

**Table S11**. Models from Stage 2 analyses explaining variation in response variables, ranked by AICc. Model selection results are presented as in Table S10.

| **log(Mass Gain/hr)** | ***k*** | **ΔAICc** | **ωi** |
| --- | --- | --- | --- |
| **Date + Logger + Age + (Age - T_1_)_+_ + Wing Loading + SST + Age*SST +**  **(Age - T_1_)_+_*SST** | **12** | **0.00** | **0.98** |
| Date + Logger + Age + (Age - T_1_)_+_ + Wing Loading + SST | 10 | 8.81 | 0.01 |
| Date + Logger + Age + (Age - T_1_)_+_ + SST + Age*SST + (Age - T_1_)_+_*SST | 11 | 9.73 | 0.01 |
| Date + Logger + Age + (Age - T_1_)_+_ + Wing Loading + Cloud Cover | 10 | 13.72 | 0.00 |
| Date + Logger + Age + (Age - T_1_)_+_ + Wing Loading + Cloud Cover +  Age*Cloud Cover + (Age - T_1_)_+_*Cloud Cover | 12 | 15.25 | 0.00 |
| Date + Logger + Age + (Age - T_1_)_+_ + SST | 9 | 17.44 | 0.00 |
| Date + Logger + Age + (Age - T_1_)_+_ + Wing Loading | 9 | 17.63 | 0.00 |
| Date + Logger + Age + (Age - T_1_)_+_ + Cloud Cover | 9 | 18.36 | 0.00 |
| Date + Logger + Age + (Age - T_1_)_+_ + Wing Loading + Med. Date | 10 | 19.71 | 0.00 |
| Date + Logger + Age + (Age - T_1_)_+_ + Cloud Cover + Age*Cloud Cover +  (Age - T_1_)_+_*Cloud Cover | 11 | 20.12 | 0.00 |
| Date + Logger + Age + (Age - T_1_)_+_ + Wing Loading + Med. Date + Age*Med. Date + (Age - T_1_)_+_* Med. Date | 12 | 22.82 | 0.00 |
| Date + Logger + Age + (Age - T_1_)_+_ | 8 | 23.49 | 0.00 |
| Date + Logger + Age + (Age - T_1_)_+_ + Med. Date | 9 | 25.09 | 0.00 |
| Date + Logger + Age + (Age - T_1_)_+_ + Med. Date + Logger + Age*Med. Date +  (Age - T_1_)_+_* Med. Date | 11 | 28.48 | 0.00 |
| **Mass Gain** | ***k*** | **ΔAICc** | **ωi** |
| **Date + Logger + Age + (Age - T_1_)_+_ + Wing Loading + SST** | **10** | **0.00** | **0.48** |
| Date + Logger + Age + (Age - T_1_)_+_ + Wing Loading + SST + Age*SST +  (Age - T_1_)_+_*SST | 12 | 0.38 | 0.40 |
| Date + Logger + Age + (Age - T_1_)_+_ + Wing Loading + Med. Date + Age*Med. Date + (Age - T_1_)_+_*Med. Date | 12 | 4.14 | 0.06 |
| Date + Logger + Age + (Age - T_1_)_+_ + Wing Loading + Cloud Cover + Age*Cloud Cover + (Age - T1)+*Cloud Cover | 12 | 6.13 | 0.02 |
| Date + Logger + Age + (Age - T_1_)_+_ + Wing Loading | 9 | 6.66 | 0.02 |
| Date + Logger + Age + (Age - T_1_)_+_ + Wing Loading + Med. Date | 10 | 7.45 | 0.01 |
| Date + Logger + Age + (Age - T_1_)_+_ + Wing Loading + Cloud Cover | 10 | 8.67 | 0.01 |
| Date + Logger + Age + (Age - T_1_)_+_ + Med. Date + Age* Med. Date +  (Age - T_1_)_+_*Med. Date | 11 | 54.04 | 0.00 |
| Date + Logger + Age + (Age - T_1_)_+_ + SST | 9 | 56.13 | 0.00 |
| Date + Logger + Age + (Age - T_1_)_+_ + Cloud Cover + Age* Cloud Cover +  (Age - T_1_)_+_*Cloud Cover | 11 | 56.70 | 0.00 |
| Date + Logger + Age + (Age - T_1_)_+_ + SST + Age*SST + (Age - T_1_)_+_*SST | 11 | 56.70 | 0.00 |
| Date + Logger + Age + (Age - T_1_)_+_ + Med. Date | 9 | 56.86 | 0.00 |
| Date + Logger + Age + (Age - T_1_)_+_ | 8 | 57.82 | 0.00 |
| Date + Logger + Age + (Age - T_1_)_+_ + Cloud Cover | 9 | 59.67 | 0.00 |
| **sqrt(Absence Duration)** | ***k*** | **ΔAICc** | **ωi** |
| **Date + Logger + Age + Age^2^ + Wing Loading + SST** | **10** | **0.00** | **0.66** |
| Date + Logger + Age + Age^2^ + Wing Loading + SST + Age*SST + Age^2^*SST | 12 | 1.33 | 0.34 |
| Date + Logger + Age + Age^2^ + Wing Loading | 9 | 13.13 | 0.00 |
| Date + Logger + Age + Age^2^ + Wing Loading + Med. Date | 10 | 15.20 | 0.00 |
| Date + Logger + Age + Age^2^ + Wing Loading + Cloud Cover | 10 | 15.21 | 0.00 |
| Date + Logger + Age + Age^2^ + Wing Loading + Cloud Cover + Age*Cloud Cover + Age^2^*Cloud Cover | 12 | 15.92 | 0.00 |
| Date + Logger + Age + Age^2^ + Wing Loading + Med. Date + Age*Med. Date +  Age^2^*Med. Date | 12 | 17.16 | 0.00 |
| Date + Logger + Age + Age^2^ + Med. Date | 9 | 48.79 | 0.00 |
| Date + Logger + Age + Age^2^ + SST | 9 | 49.49 | 0.00 |
| Date + Logger + Age + Age^2^ + SST + Age*SST + Age^2^*SST | 11 | 51.10 | 0.00 |
| Date + Logger + Age + Age^2^ + Med. Date + Age* Med. Date + Age^2^* Med. Date | 11 | 54.77 | 0.00 |
| Date + Logger + Age + Age^2^ | 8 | 55.48 | 0.00 |
| Date + Logger + Age + Age^2^ + Cloud Cover | 9 | 57.55 | 0.00 |
| Date + Logger + Age + Age^2^ + Cloud Cover + Age*Cloud Cover +  Age^2^*Cloud Cover | 11 | 59.09 | 0.00 |
| **sqrt(Total Distance)** | ***k*** | **ΔAICc** | **ωi** |
| **Date + Logger + Age + Age^2^ + Wing Loading + SST** | **10** | **0.00** | **0.85** |
| Date + Logger + Age + Age^2^ + Wing Loading + SST + Age*SST + Age^2^*SST | 12 | 3.46 | 0.15 |
| Date + Logger + Age + Age^2^ + Wing Loading | 9 | 13.16 | 0.00 |
| Date + Logger + Age + Age^2^ + Wing Loading + Cloud Cover + Age*Cloud Cover + Age^2^*Cloud Cover | 12 | 14.24 | 0.00 |
| Date + Logger + Age + Age^2^ + Wing Loading + Med. Date | 10 | 14.71 | 0.00 |
| Date + Logger + Age + Age^2^ + Wing Loading + Cloud Cover | 10 | 14.83 | 0.00 |
| Date + Logger + Age + Age^2^ + Wing Loading + Med. Date + Age*Med. Date +  Age^2^*Med. Date | 12 | 18.86 | 0.00 |
| Date + Logger + Age + Age^2^ + SST | 9 | 35.89 | 0.00 |
| Date + Logger + Age + Age^2^ + SST + Age*SST + Age^2^*SST | 11 | 39.51 | 0.00 |
| Date + Logger + Age + Age^2^ + Med. Date | 9 | 42.92 | 0.00 |
| Date + Logger + Age + Age^2^ | 8 | 44.66 | 0.00 |
| Date + Logger + Age + Age^2^ + Cloud Cover + Age*Cloud Cover + Age^2^*Cloud Cover | 11 | 45.89 | 0.00 |
| Date + Logger + Age + Age^2^ + Cloud Cover | 9 | 46.13 | 0.00 |
| Date + Logger + Age + Age^2^ + Age*Med. Date + Age^2^*Med. Date | 11 | 46.78 | 0.00 |
| **sqrt(Time Searching)** | ***k*** | **ΔAICc** | **ωi** |
| **Date + Logger + Age + (Age - T_1_)_+_ + Wing Loading + SST** | **10** | **0.00** | **0.60** |
| Date + Logger + Age + (Age - T_1_)_+_ + Wing Loading + SST + Age*SST +  (Age - T_1_)_+_*SST | 12 | 2.83 | 0.15 |
| Date + Logger + Age + (Age - T_1_)_+_ + Wing Loading | 9 | 3.74 | 0.09 |
| Date + Logger + Age + (Age - T_1_)_+_ + Wing Loading + Med. Date | 10 | 3.86 | 0.09 |
| Date + Logger + Age + (Age - T_1_)_+_ + Wing Loading + Cloud Cover | 10 | 5.37 | 0.04 |
| Date + Logger + Age + (Age - T_1_)_+_ + Wing Loading + Med. Date + Age*Med. Date + (Age - T_1_)_+_*Med. Date | 12 | 6.74 | 0.02 |
| Date + Logger + Age + (Age - T_1_)_+_ + Wing Loading + Cloud Cover +  Age*Cloud Cover + (Age - T_1_)_+_*Cloud Cover | 12 | 8.62 | 0.01 |
| Date + Logger + Age + (Age - T_1_)_+_ + Med. Date | 9 | 51.75 | 0.00 |
| Date + Logger + Age + (Age - T_1_)_+_ + Med. Date + Age*Med. Date +  (Age - T_1_)_+_*Med. Date | 11 | 54.09 | 0.00 |
| Date + Logger + Age + (Age - T_1_)_+_ + SST | 9 | 61.40 | 0.00 |
| Date + Logger + Age + (Age - T_1_)_+_ | 8 | 61.54 | 0.00 |
| Date + Logger + Age + (Age - T_1_)_+_ + Cloud Cover | 9 | 62.56 | 0.00 |
| Date + Logger + Age + (Age - T_1_)_+_ + SST + Age*SST + (Age - T_1_)_+_*SST | 11 | 64.19 | 0.00 |
| Date + Logger + Age + (Age - T_1_)_+_ + Cloud Cover + Age*Cloud Cover +  (Age - T_1_)_+_*Cloud Cover | 11 | 66.68 | 0.00 |

## 14. Spatial Segregation by Age, Sex, and Breeding Season

**Table S12.** Observed and randomized spatial overlap (BA) of core (50%) and general use (95%) utilization distributions of Nazca boobies while foraging, compared among AgeGroups for each Sex, across all Breeding Seasons. Y = Young; M = Middle Age; O = Old; O+ = Oldest. The significance of each P value was corrected for multiple comparisons to reduce Type I error. Significance (P<α_crit_; Benjamini and Hochberg 1995) denoted in bold.

|  |  | 95% |  |  |  | 50% |  | |
| --- | --- | --- | --- | --- | --- | --- | --- | --- |
|  | **Observed** | **Randomized** | **P** |  | **Observed** | **Randomized** | | **P** |
| **Sex** |  |  |  |  |  |  | |  |
| Males vs. Females | **0.89** | **0.91 ± 0.00** | **<0.001** |  | **0.50** | **0.47 ± 0.01** | | **<0.001** |
| **Males** |  |  |  |  |  |  | |  |
| Y vs. M | 0.88 | 0.90 ± 0.01 | 0.275 |  | 0.46 | 0.45 ± 0.02 | | 0.692 |
| Y vs. O | 0.86 | 0.88 ± 0.01 | 0.123 |  | 0.45 | 0.44 ± 0.02 | | 0.693 |
| Y vs. O+ | 0.84 | 0.85 ± 0.02 | 0.501 |  | 0.43 | 0.41 ± 0.03 | | 0.634 |
| M vs. O | 0.90 | 0.89 ± 0.01 | 0.946 |  | 0.45 | 0.45 ± 0.02 | | 0.551 |
| M vs. O+ | 0.84 | 0.85 ± 0.02 | 0.334 |  | 0.41 | 0.42 ± 0.03 | | 0.384 |
| O vs. O+ | 0.86 | 0.84 ± 0.02 | 0.825 |  | 0.43 | 0.41 ± 0.03 | | 0.753 |
| **Females** |  |  |  |  |  |  | |  |
| Y vs. M | **0.83** | **0.87 ± 0.01** | **0.002** |  | 0.41 | 0.44 ± 0.01 | | 0.021 |
| Y vs. O | 0.84 | 0.87 ± 0.01 | 0.051 |  | 0.41 | 0.44 ± 0.02 | | 0.014 |
| Y vs. O+ | 0.78 | 0.83 ± 0.02 | 0.019 |  | 0.36 | 0.41 ± 0.03 | | 0.038 |
| M vs. O | 0.86 | 0.87 ± 0.01 | 0.310 |  | 0.43 | 0.44 ± 0.02 | | 0.271 |
| M vs. O+ | 0.86 | 0.82 ± 0.02 | 0.963 |  | 0.43 | 0.41 ± 0.03 | | 0.715 |
| O vs. O+ | 0.84 | 0.82 ± 0.02 | 0.783 |  | 0.42 | 0.41 ± 0.03 | | 0.537 |

**Table S13.** Observed and randomized spatial overlap (Bhattacharyya’s affinity, BA) of core (50%) and general use (95%) utilization distributions of Nazca boobies during localized searching, compared among Breeding Seasons for each Sex. Results presented as in Table S12.

|  |  | 95% |  |  |  | 50% |  |
| --- | --- | --- | --- | --- | --- | --- | --- |
|  | **Observed** | **Randomized** | **P** |  | **Observed** | **Randomized** | **P** |
| **Males** |  |  |  |  |  |  |  |
| 2011 vs. 2012 | 0.82 | 0.83 ± 0.02 | 0.158 |  | 0.41 | 0.41 ± 0.02 | 0.429 |
| 2011 vs. 2014 | **0.77** | **0.83 ± 0.02** | **0.006** |  | **0.30** | **0.41 ± 0.03** | **0.001** |
| 2011 vs. 2015 | **0.68** | **0.84 ± 0.01** | **<0.001** |  | **0.34** | **0.42 ± 0.02** | **<0.001** |
| 2011 vs. 2016 | **0.77** | **0.85 ± 0.01** | **<0.001** |  | **0.33** | **0.43 ± 0.02** | **<0.001** |
| 2012 vs. 2014 | 0.77 | 0.81 ± 0.02 | 0.101 |  | 0.35 | 0.39 ± 0.03 | 0.083 |
| 2012 vs. 2015 | **0.62** | **0.82 ± 0.02** | **<0.001** |  | **0.28** | **0.40 ± 0.03** | **<0.001** |
| 2012 vs. 2016 | **0.78** | **0.83 ± 0.02** | **0.008** |  | **0.35** | **0.41 ± 0.02** | **0.014** |
| 2014 vs. 2015 | **0.66** | **0.81 ± 0.02** | **<0.001** |  | **0.14** | **0.40 ± 0.03** | **<0.001** |
| 2014 vs. 2016 | **0.70** | **0.82 ± 0.02** | **<0.001** |  | **0.26** | **0.40 ± 0.03** | **<0.001** |
| 2015 vs. 2016 | **0.55** | **0.84 ± 0.02** | **<0.001** |  | **0.23** | **0.41 ± 0.02** | **<0.001** |
| **Females** |  |  |  |  |  |  |  |
| 2011 vs. 2012 | 0.81 | 0.82 ± 0.02 | 0.292 |  | 0.42 | 0.42 ± 0.02 | 0.535 |
| 2011 vs. 2014 | 0.81 | 0.81 ± 0.02 | 0.412 |  | **0.34** | **0.41 ± 0.02** | **0.006** |
| 2011 vs. 2015 | **0.72** | **0.83 ± 0.02** | **<0.001** |  | **0.33** | **0.42 ± 0.02** | **<0.001** |
| 2011 vs. 2016 | **0.72** | **0.84 ± 0.02** | **<0.001** |  | **0.30** | **0.43 ± 0.02** | **<0.001** |
| 2012 vs. 2014 | 0.80 | 0.79 ± 0.02 | 0.615 |  | 0.35 | 0.40 ± 0.03 | 0.072 |
| 2012 vs. 2015 | **0.70** | **0.80 ± 0.02** | **<0.001** |  | **0.28** | **0.41 ± 0.02** | **<0.001** |
| 2012 vs. 2016 | **0.66** | **0.81 ± 0.02** | **<0.001** |  | **0.28** | **0.41 ± 0.02** | **<0.001** |
| 2014 vs. 2015 | **0.64** | **0.80 ± 0.02** | **<0.001** |  | **0.15** | **0.40 ± 0.03** | **<0.001** |
| 2014 vs. 2016 | **0.68** | **0.80 ± 0.02** | **<0.001** |  | **0.26** | **0.41 ± 0.03** | **<0.001** |
| 2015 vs. 2016 | **0.57** | **0.82 ± 0.02** | **<0.001** |  | **0.21** | **0.42 ± 0.02** | **<0.001** |

**Table S14.** For each Breeding Season, observed and randomized spatial overlap (Bhattacharyya’s affinity, BA) of core (50%) and general use (95%) utilization distributions of Nazca boobies during localized searching, compared among AgeGroups for each Sex. GPS deployments returned data for only three Oldest male and four Oldest female Nazca boobies in 2011 and only one Oldest male and two Oldest female Nazca boobies in 2012, precluding pairwise comparisons between the Oldest and other age classes. Results presented as in Table S12.

| **Season** |  |  |  | **Males** |  |  |  |  |  |  | **Females** |  |  |  |  |  |
| --- | --- | --- | --- | --- | --- | --- | --- | --- | --- | --- | --- | --- | --- | --- | --- | --- |
|  |  |  | **95%** |  |  |  | **50%** |  |  |  | **95%** |  |  |  | **50%** |  |
|  | **Age Groups** | **Obs.** | **Rand.** | **P** |  | **Obs.** | **Rand.** | **P** |  | **Obs.** | **Rand.** | **P** |  | **Obs.** | **Rand.** | **P** |
| **2011** | Y vs. M | 0.83 | 0.87 ± 0.02 | 0.073 |  | 0.39 | 0.40 ± 0.03 | 0.345 |  | 0.83 | 0.82 ± 0.03 | 0.565 |  | 0.45 | 0.42 ± 0.03 | 0.945 |
|  | Y vs. O | 0.88 | 0.86 ± 0.02 | 0.834 |  | 0.36 | 0.39 ± 0.04 | 0.199 |  | 0.83 | 0.81 ± 0.03 | 0.632 |  | 0.41 | 0.41 ± 0.03 | 0.405 |
|  | Y vs. O+ | - | - | - |  | - | - | - |  | - | - | - |  | - | - | - |
|  | M vs. O | 0.86 | 0.86 ± 0.02 | 0.560 |  | 0.41 | 0.39 ± 0.04 | 0.685 |  | 0.85 | 0.81 ± 0.03 | 0.921 |  | 0.43 | 0.41 ± 0.03 | 0.617 |
|  | M vs. O+ | - | - | - |  | - | - | - |  | **-** | **-** | **-** |  | **-** | **-** | **-** |
|  | O vs. O+ | - | - | - |  | - | - | - |  | **-** | **-** | **-** |  | **-** | **-** | **-** |
|  | Y vs. M | 0.83 | 0.79 ± 0.03 | 0.879 |  | 0.36 | 0.38 ± 0.05 | 0.319 |  | 0.68 | 0.74 ± 0.05 | 0.101 |  | 0.29 | 0.33 ± 0.05 | 0.188 |
|  | Y vs. O | 0.76 | 0.79 ± 0.03 | 0.153 |  | 0.38 | 0.37 ± 0.04 | 0.567 |  | 0.82 | 0.76 ± 0.04 | 0.950 |  | 0.31 | 0.35 ± 0.05 | 0.158 |
|  | Y vs. O+ | - | - | - |  | - | - | - |  | - | - | - |  | - | - | - |
| **2012** | M vs. O | 0.82 | 0.79 ± 0.04 | 0.824 |  | 0.41 | 0.37 ± 0.05 | 0.766 |  | 0.74 | 0.73 ± 0.05 | 0.521 |  | 0.41 | 0.32 ± 0.06 | 0.971 |
|  | M vs. O+ | - | - | - |  | - | - | - |  | - | - | - |  | - | - | - |
|  | O vs. O+ | - | - | - |  | - | - | - |  | - | - | - |  | - | - | - |
| **2014** | Y vs. M | 0.77 | 0.78 ± 0.04 | 0.322 |  | 0.38 | 0.36 ± 0.05 | 0.672 |  | 0.71 | 0.75 ± 0.05 | 0.187 |  | 0.39 | 0.33 ± 0.05 | 0.870 |
|  | Y vs. O | 0.76 | 0.77 ± 0.04 | 0.377 |  | 0.22 | 0.35 ± 0.05 | 0.019 |  | 0.72 | 0.75 ± 0.04 | 0.251 |  | 0.34 | 0.33 ± 0.05 | 0.544 |
|  | Y vs. O+ | 0.72 | 0.74 ± 0.06 | 0.369 |  | 0.32 | 0.31 ± 0.07 | 0.540 |  | 0.63 | 0.69 ± 0.07 | 0.163 |  | 0.22 | 0.28 ± 0.07 | 0.206 |
|  | M vs. O | 0.76 | 0.78 ± 0.04 | 0.261 |  | **0.18** | **0.35 ± 0.05** | **0.003** |  | 0.82 | 0.76 ± 0.04 | 0.931 |  | 0.40 | 0.35 ± 0.05 | 0.901 |
|  | M vs. O+ | 0.68 | 0.74 ± 0.06 | 0.130 |  | 0.28 | 0.31 ± 0.06 | 0.275 |  | 0.65 | 0.70 ± 0.07 | 0.195 |  | 0.20 | 0.29 ± 0.07 | 0.112 |
|  | O vs. O+ | 0.74 | 0.74 ± 0.06 | 0.438 |  | 0.20 | 0.30 ± 0.07 | 0.084 |  | 0.76 | 0.71 ± 0.07 | 0.942 |  | 0.24 | 0.29 ± 0.07 | 0.204 |
| **2015** | Y vs. M | 0.85 | 0.76 ± 0.05 | 0.991 |  | 0.38 | 0.35 ± 0.04 | 0.710 |  | 0.66 | 0.73 ± 0.05 | 0.069 |  | 0.29 | 0.36 ± 0.04 | 0.068 |
|  | Y vs. O | 0.72 | 0.76 ± 0.05 | 0.216 |  | 0.33 | 0.35 ± 0.04 | 0.322 |  | 0.74 | 0.72 ± 0.05 | 0.634 |  | 0.29 | 0.35 ± 0.05 | 0.137 |
|  | Y vs. O+ | 0.73 | 0.74 ± 0.05 | 0.363 |  | 0.34 | 0.33 ± 0.05 | 0.500 |  | 0.55 | 0.70 ± 0.06 | 0.014 |  | 0.26 | 0.33 ± 0.05 | 0.118 |
|  | M vs. O | 0.74 | 0.76 ± 0.05 | 0.291 |  | 0.32 | 0.35 ± 0.04 | 0.229 |  | 0.74 | 0.72 ± 0.05 | 0.624 |  | 0.39 | 0.35 ± 0.05 | 0.847 |
|  | M vs. O+ | 0.74 | 0.75 ± 0.05 | 0.425 |  | 0.33 | 0.34 ± 0.05 | 0.374 |  | 0.66 | 0.70 ± 0.05 | 0.182 |  | 0.33 | 0.33 ± 0.06 | 0.452 |
|  | O vs. O+ | 0.76 | 0.75 ± 0.05 | 0.527 |  | 0.33 | 0.33 ± 0.05 | 0.379 |  | 0.68 | 0.69 ± 0.06 | 0.395 |  | 0.29 | 0.32 ± 0.06 | 0.285 |
| **2016** | Y vs. M | 0.80 | 0.82 ± 0.03 | 0.205 |  | **0.22** | **0.36 ± 0.05** | **0.007** |  | 0.77 | 0.76 ± 0.04 | 0.610 |  | 0.13 | 0.24 ± 0.06 | 0.031 |
|  | Y vs. O | 0.74 | 0.79 ± 0.04 | 0.098 |  | 0.20 | 0.33 ± 0.06 | 0.027 |  | 0.70 | 0.73 ± 0.05 | 0.196 |  | **0.08** | **0.22 ± 0.06** | **0.016** |
|  | Y vs. O+ | **0.60** | **0.76 ± 0.05** | **0.002** |  | 0.16 | 0.29 ± 0.06 | 0.024 |  | **0.56** | **0.72 ± 0.05** | **0.007** |  | **0.03** | **0.22 ± 0.06** | **<0.001** |
|  | M vs. O | 0.84 | 0.83 ± 0.03 | 0.577 |  | 0.42 | 0.37 ± 0.04 | 0.919 |  | 0.73 | 0.75 ± 0.04 | 0.221 |  | 0.15 | 0.24 ± 0.06 | 0.076 |
|  | M vs. O+ | 0.79 | 0.80 ± 0.04 | 0.337 |  | 0.39 | 0.33 ± 0.05 | 0.872 |  | 0.69 | 0.74 ± 0.05 | 0.131 |  | 0.21 | 0.23 ± 0.06 | 0.388 |
|  | O vs. O+ | 0.80 | 0.77 ± 0.04 | 0.683 |  | 0.37 | 0.32 ± 0.06 | 0.752 |  | 0.72 | 0.72 ± 0.05 | 0.442 |  | 0.32 | 0.21 ± 0.06 | 0.972 |

## 15. Foraging Tracks by Breeding Season

**
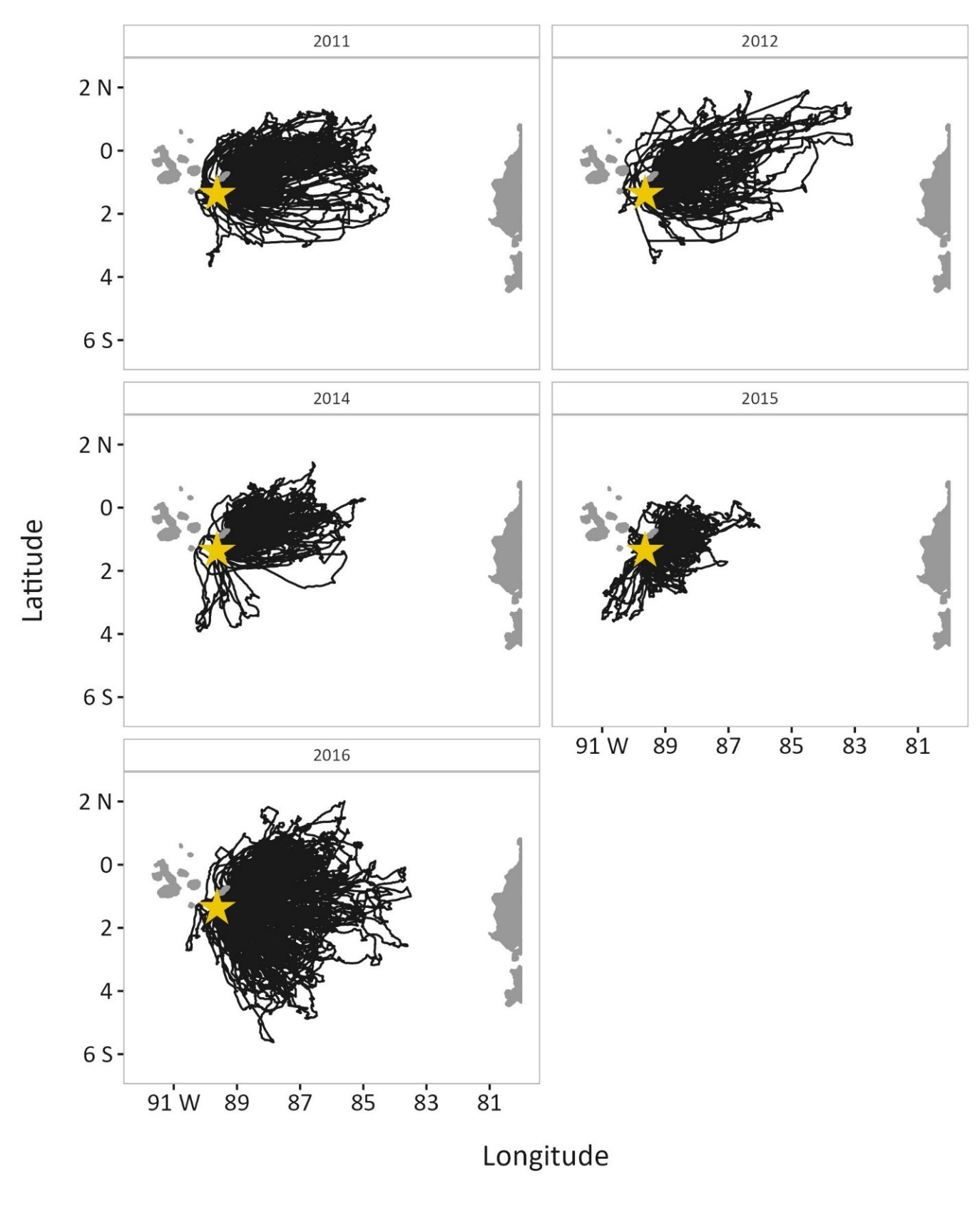
**

**Figure S6.** Foraging routes by breeding season for Nazca boobies during egg incubation from the breeding colony at Punta Cevallos, Isla Española (colony marked by a yellow star at 1°23’S, 89°37’W).

## 16. Mass at Departure and Arrival in Nazca Boobies

**Table S15.** Mass at departure (kg) and Mass at arrival (kg) by AgeGroup for male and female Nazca boobies included in this study. Sample size (N), mean, and SD are shown for each AgeGroup and sex combination.

|  | **Sex** | **AgeGroup** | **N** | **Mean** | **SD** |
| --- | --- | --- | --- | --- | --- |
| **Departure**  **Mass** | F | Young | 151 | 1.92 | 0.18 |
|  |  | Middle Age | 102 | 1.89 | 0.15 |
|  |  | Old | 156 | 1.94 | 0.17 |
|  |  | Oldest | 52 | 1.82 | 0.12 |
|  | M | Young | 148 | 1.63 | 0.13 |
|  |  | Middle Age | 126 | 1.62 | 0.15 |
|  |  | Old | 171 | 1.68 | 0.16 |
|  |  | Oldest | 47 | 1.61 | 0.12 |
| **Arrival**  **Mass** | F | Young | 151 | 2.31 | 0.21 |
|  |  | Middle Age | 102 | 2.30 | 0.19 |
|  |  | Old | 156 | 2.32 | 0.20 |
|  |  | Oldest | 52 | 2.22 | 0.17 |
|  | M | Young | 148 | 1.98 | 0.16 |
|  |  | Middle Age | 126 | 1.95 | 0.17 |
|  |  | Old | 171 | 1.98 | 0.19 |
|  |  | Oldest | 47 | 1.92 | 0.13 |

# LITERATURE CITED

Benjamini, Y., and Y. Hochberg (1995). Controlling the false discovery rate: a practical and powerful approach to multiple testing. Journal of the Royal Statistical Society. Series B (Methodological) 57:289–300.

Bivand, R., and N. Lewin-Koh (2021). maptools: tools for handling spatial objects. R package version 1.1-1. Available at https://cran.r-project.org/package=maptools.

Burnham, K. P., and D. R. Anderson (2010). Model Selection and Multimodel Inference: A Practical Information-Theoretic Approach. 2nd edition. Springer, New York.

Chamberlain, S. (2016). rerddap: General purpose client for ‘ERDDAP’servers. R package version 0.3. 4. [Online.] Available at https://github.com/ropensci/rerddap.

Cherel, Y., J.-P. Robin, and Y. Le Maho (1988). Physiology and biochemistry of long-term fasting in birds. Canadian Journal of Zoology 66:159–166.

Hedd, A., R. Gales, and N. Brothers (2001). Foraging strategies of shy albatross *Thalassarche cauta* breeding at Albatross Island, Tasmania, Australia. Marine Ecology Progress Series 224:267–282.

Howard, J. L., E. M. Tompkins, and D. J. Anderson (2021). Effects of age, sex, and ENSO phase on foraging and flight performance in Nazca boobies. Ecology and Evolution 11:4084–4100.

Lerma, M., J. Serratosa, G. Luna-Jorquera, and S. Garthe (2020). Foraging ecology of masked boobies (*Sula dactylatra*) in the world’s largest “oceanic desert.” Marine Biology 167:87.

Mendez, L., C. Cotté, A. Prudor, and H. Weimerskirch (2016). Variability in foraging behaviour of red-footed boobies nesting on Europa Island. Acta Oecologica 72:87–97.

Noonan, M. J., C. H. Fleming, T. S. Akre, J. Drescher-lehman, E. Gurarie, R. Kays, and J. M. Calabrese (2019). The fast and the spurious: scale-free estimation of speed and distance traveled from animal tracking data. Movement Ecology 7:1–15.

Palacios, D. M. (2004). Seasonal patterns of sea-surface temperature and ocean color around the Galápagos: Regional and local influences. Deep-Sea Research Part II: Topical Studies in Oceanography 51:43–57.

Prince, P. A., C. Ricketts, and G. Thomas (1981). Weight loss in incubating albatrosses and its implications for their energy and food requirements. The Condor 83:238–242.

Santoso, A., M. J. Mcphaden, and W. Cai (2017). The defining characteristics of ENSO extremes and the strong 2015/2016 El Niño. Reviews of Geophysics 55:1079–1129.

Zavalaga, C. B., S. D. Emslie, F. A. Estela, M. S. Müller, G. Dell’Omo, D. J. Anderson, M. S. Muller, G. D. Omo, and D. J. Anderson (2012). Overnight foraging trips by chick-rearing Nazca boobies *Sula granti* and the risk of attack by predatory fish. Ibis 154:61–73.
